# Potential for DNA-based identification of Great Lakes fauna: match and mismatch between taxa inventories and DNA barcode libraries

**DOI:** 10.1038/srep12162

**Published:** 2015-07-22

**Authors:** Anett S. Trebitz, Joel C. Hoffman, George W. Grant, Tyler M. Billehus, Erik M. Pilgrim

**Affiliations:** 1 U.S. Environmental Protection Agency, National Health and Environmental Effects Research Laboratory, Duluth, Minnesota, USA; 2 U.S. Environmental Protection Agency, National Exposure Research Laboratory, Cincinnati Ohio, USA

## Abstract

DNA-based identification of mixed-organism samples offers the potential to greatly reduce the need for resource-intensive morphological identification, which would be of value both to bioassessment and non-native species monitoring. The ability to assign species identities to DNA sequences found depends on the availability of comprehensive DNA reference libraries. Here, we compile inventories for aquatic metazoans extant in or threatening to invade the Laurentian Great Lakes and examine the availability of reference mitochondrial COI DNA sequences (barcodes) in the Barcode of Life Data System for them. We found barcode libraries largely complete for extant and threatening-to-invade vertebrates (100% of reptile, 99% of fish, and 92% of amphibian species had barcodes). In contrast, barcode libraries remain poorly developed for precisely those organisms where morphological identification is most challenging; 46% of extant invertebrates lacked reference barcodes with rates especially high among rotifers, oligochaetes, and mites. Lack of species-level identification for many aquatic invertebrates also is a barrier to matching DNA sequences with physical specimens. Attaining the potential for DNA-based identification of mixed-organism samples covering the breadth of aquatic fauna requires a concerted effort to build supporting barcode libraries and voucher collections.

The rapidly increasing capacity and decreasing expense of DNA sequencing technology offers the potential to supplant the need for morphological identification of organisms^1^,^2^. Morphological identification can require considerable time, resources, and expertise, particularly for taxa that are species rich, require microscopy to identify, and where samples include extensive bycatch (debris, non-target taxa) from which the organisms of interest must be separated. Zooplankton and benthic macroinvertebrates are notable examples of aquatic taxa that are important to biotic community assessment (“bioassessment”, hereafter) yet labor-intensive to enumerate and for which DNA-based identification technology is therefore of considerable interest^3^,^4^.

Current efforts for developing DNA-based identification technology of aquatic samples fall into two general approaches. One approach focuses on determining presence or absence of preselected species using primers that bind to short species-specific DNA fragments shed into the environment^5^. This approach offers the potential for rapid feedback concerning presence of the target species but provides no information concerning the rest of the community, and is thus most relevant for species of predetermined concern (e.g., Asian carp threatening to invade the Great Lakes^6^). The second, taxonomically broader approach seeks to determine community composition by running longer DNA segments amplified from water or mixed-organism tissue samples through a massively parallel DNA sequencing followed by bioinformatics processing to generate a list of species present^7^,^8^. With this approach, hereafter referred to as metabarcoding (also “metagenomics” and “environmental barcoding” in the literature), the sequences obtained are clustered into operational taxonomic units (OTUs) based on genetic distance; taxonomic labels are then assigned to those OTUs by matching the sequences to DNA barcodes for known (i.e., morphologically identified) specimens. The Consortium for the Barcode of Life exists for the express purpose of fostering the development of the necessary barcode reference libraries, and an on-line database and informatics workbench known as the Barcode of Life Data System (BOLD; < www.barcodinglife.com>) has emerged as a central resource via which DNA barcode information is assembled, documented, and disseminated^9^,^10^.

Our focus is the metabarcoding approach because of its potential for characterizing biological composition as well as detecting a broad suite of non-native species. We focus specifically on the applicability of metabarcoding to identifying aquatic fauna of the Laurentian Great Lakes (North America). The Great Lakes are an expansive (~244,000 km^2^ surface area, 17,000 km of shoreline, holding ~20% of the world’s fresh water) and environmentally complex set of water bodies which support not only a diversity of fauna and flora, but also a human population of >30 million whose recreation and commerce depend heavily on the lakes and whose activities result in significant anthropogenic stress to the lakes^11^,^12^,^13^. In the Great Lakes as in other water body types, bioassessment forms the basis for evaluating ecological status and trends^14^,^15^. Initiatives are also underway to monitor for an array of non-native species, whose arrival and impacts continue to threaten the Great Lakes ecological condition and economy^16^,^17^.

Our objective here is to assess the degree to which reference barcodes are available for aquatic fauna currently found in or deemed likely to invade the Great Lakes. The completeness of reference barcode libraries determines how likely it is that a species (whether native or introduced) will be detected based on sequences recovered from a mixed-organism sample, and more broadly, how much work remains to make metabarcoding a viable tool to support bioassessment and non-native species monitoring^18^. We focus on metazoan taxa (reptiles, amphibians, fishes, zooplankton, and benthic macroinvertebrates) as these are the groups of most interest in Great Lakes bioassessment and non-native species monitoring and best documented in the BOLD database (as opposed to fungi, diatoms, protozoans). These metazoan groups exhibit diversity not only taxonomically but in traits such as body size, life history, and habitat occupied that make comprehensive assessments nontrivial. Zooplankton and benthic macroinvertebrate samples routinely require labor-intensive laboratory processing to pick, sort, and enumerate, and morphological identification of some species requires extensive preparation (e.g., slide mounts) and expertise^19^,^20^. Adults of the vertebrates are ordinarily identifiable in the field, but more difficult to identify life stages such as eggs and larvae are also of monitoring interest. Metabarcoding offers potential for efficiency in monitoring for all these groups^21^,^22^,^23^ and makes species-level identification possible where morphological identification fails – e.g., for immature life-stages, damaged and partial specimens, and morphologically “cryptic” species^24^. The DNA marker we focus on is mitochondrial cytochrome c oxidase subunit I (COI), which has been proposed as a “universal” barcoding locus for animals and is what the BOLD database compiles^9^. We recognize that the COI barcode does not successfully resolve all animal taxa and that there are other markers in use^2^,^7^, but their discussion is outside the scope of this study.

Our geographic focus is waters of the five Laurentian Great Lakes proper (Lakes Erie, Huron, Michigan, Ontario, and Superior) including their connecting channels and the smaller aquatic ecosystems connected to the lakes via bi-directional water exchange (e.g., coastal wetlands, embayments, terminal river reaches). Our aim is species-level taxonomy because that is the level at which DNA-barcode based identifications are sought and invasive species monitoring must be conducted. However, we also included organisms for which the most resolved identification was at a coarser taxonomic level, to gain understanding of how data resolution issues affect the utility of metabarcoding. Species-level identification is necessary to distinguish native from non-native taxa within the same genus (Great Lakes examples include *Daphnia* waterfleas, *Pisidium* peaclams, *Notropis* fishes), but other goals of biological monitoring may be attainable with coarser-level taxonomy (e.g., biotic integrity indices often use genus or family level data^19^). Given the considerable geographic scope of the Great Lakes and the breadth of organism groups we consider, we expect our findings to be broadly illustrative of the current capacity of barcode libraries to support metabarcoding of freshwater aquatic fauna.

## Results

### Inventory composition and taxonomic resolution

We compiled lists of >1600 aquatic metazoans currently found in the Great Lakes (Table 1) and >100 aquatic metazoans considered invasion threats to the Great Lakes (Table 2). Extant vertebrates include 181 species of fishes of which 37 (20%) are introduced rather than native, and 15 species of anurans, 20 salamanders, 12 snakes, and 10 turtles, all native to the Great Lakes (the turtle *Trachemys scripta* has a non-native subspecies but we did not consider subspecies in our analysis). Extant zooplankton include 164 crustacean species (classes Branchiopoda, Maxilliopoda, and Ostracoda) of which 19 (12%) are introduced, and 201 rotifer species, all native. Extant benthic macroinvertebrates include 148 mollusks, 130 annelids (leeches and worms), 543 insects, and another 78 species of mites, malacostraca (amphipods, crayfishes, etc.), and assorted other taxa. Forty benthic invertebrate species (4%) were introduced: 18 mollusks, 7 oligochaete worms, 7 amphipods and crayfishes, 3 flatworms, 2 cnidaria, 2 insects, and 1 bryozoan.

**Figure 1:**
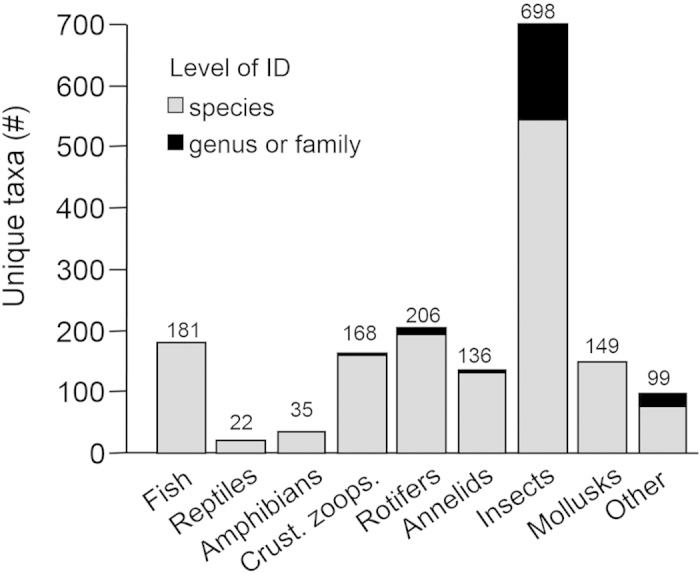
Bar graph showing distribution of extant Great Lakes aquatic fauna among taxonomic groups. Bar color denotes whether lowest level of identification is to species versus only genus or family. Taxonomic resolution is collapsed relative to the categories in Table 1: reptiles = snakes + turtles, amphibians = anurans + salamanders, crust. zoops. = branchiopods + copepods + ostracods, annelids = leeches + oligochaetes, insects are 6 groups combined, mollusks = gastropods + clams, and other = mites + crustacean benthos + other benthos.

Vertebrates were always resolved to species but there were many invertebrates that were not identified to species level in any report from Great Lakes waters (Fig. 1). Twelve zooplankton taxa (2%) were resolved only to genus, with genus-level identification most prevalent in ostracods (Table 1). Among benthic macroinvertebrates, 190 taxa (16%) were resolved only to genus and 11 (1%) were resolved only to family. Mollusks, odonates, and benthic crustacea all had species-level resolution, but coarser resolution was prevalent among mites, coleopterans, dipterans, and hemipterans (Table 1). A full list of taxa lacking species-level resolution appears in Supplementary Table S1 (online).

Unlike extant taxa, all threatening-to-invade taxa were resolved to species and were strongly biased towards vertebrates over invertebrates (98 vs. 18 species; Table 2). All but one of the vertebrates on the threatening-to-invade list were fishes; there was a single anuran and no salamanders, snakes, or turtles. Most of the invertebrates on the threatening-to-invade list were benthic crustaceans and snails; the absence of oligochaete worms and clams is conspicuous given their prominence among invertebrates already introduced to the Great Lakes.

### Barcode availability

We used the BOLD database to determine the availability of DNA barcodes for all extant and threatening-to-invade taxa that were identified to species (listed in Supplementary Table S2). What we report as barcode availability are statistics for DNA sequences meeting BOLD’s mitochondrial COI barcode standard (i.e., at least 500 base-pairs long, with <1% ambiguous bases and detailed supporting information).

**Figure 2:**
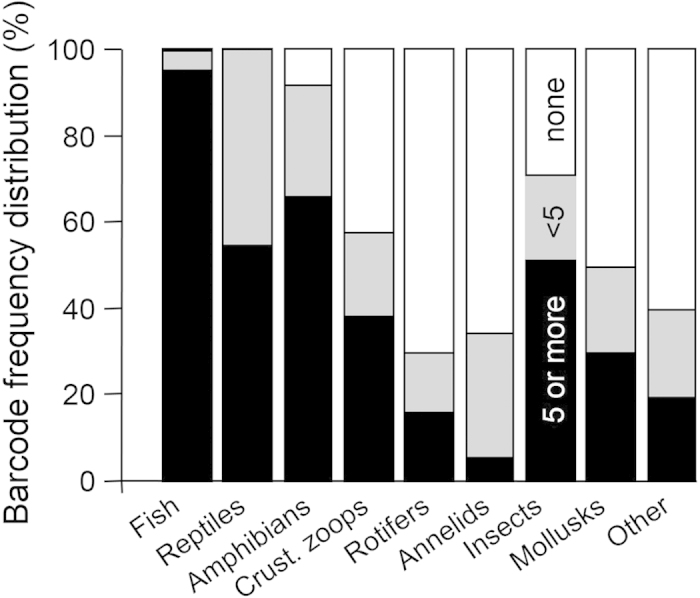
Bar graph showing availability of DNA barcodes for extant Great lakes aquatic fauna whose identity is resolved to species. The barcode availability categories from Table 1 and 2 are collapsed as follows: white = no barcodes (not listed in BOLD or zero barcodes); grey = <5 barcodes; black = 5–25 or >25 barcodes. Taxonomic groupings are as in Fig. 1.

Among extant species, barcode availability was much higher for vertebrates than invertebrates (97% vs. only 55% having at least one barcode). Every vertebrate subgroup had >90% barcode availability, whereas no invertebrate subgroup had better than 70% barcode availability (Fig. 2). All snakes and turtles had at least one barcode in BOLD and over half had five or more barcodes (our criteria for moderate capability to characterize intra-species variability); although none had over 25 barcodes (our criteria for good capability to characterize intra-species variability; Table 1). Nine percent of amphibians (anurans and salamanders) lacked barcodes in BOLD, but amphibians with barcodes tended to have more of them than the reptiles (Fig. 2, Table 1). One fish species lacked records in BOLD entirely, but most fishes had at least five barcodes and over half had >25 barcodes. Among invertebrates, barcode availability was highest for insects, crustacean zooplankton, and mollusks (all ≥50%) and lowest for rotifers and annelids (<35%; Fig. 2). Within annelids, a much higher percentage of leeches than oligochaete worms had barcodes but species in both groups generally had <5 barcodes (Table 1). Barcodes were absent entirely for the few mites that were resolved to species (Table 1). Within insects, barcode availability rates were highest for EPT taxa (order Ephemeroptera, Plecoptera, and Trichoptera) and lowest for hemipterans (true bugs) and coleopterans (beetles); EPT taxa also had the highest percentage of species with >25 barcodes (Table 2).

**Figure 3:**
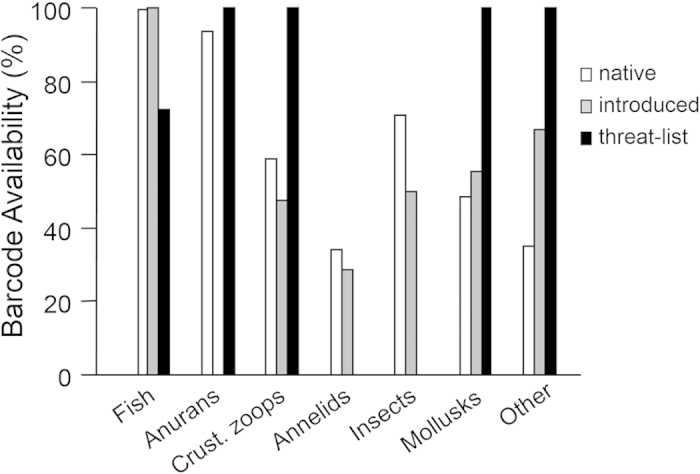
Bar graph showing percentage of native, introduced, or threatening-to-invade species having at least one DNA barcode available. Taxonomic groupings are as in Fig. 1 except that groups lacking introduced or threatening-to-invade species are omitted (i.e., no reptiles, rotifers, salamanders). Missing bars for introduced anurans and threatening-to-invade insects and annelids are because there are no such species rather than because none have barcodes.

Barcode availability rates were higher for introduced than native species of fishes and mollusks but lower for introduced than native crustacean zooplankton, annelids, and insects (Fig. 3). Fishes were the only group where 100% of the introduced species had barcodes (Fig. 3).

Threatening-to-invade fauna differed substantially from extant aquatic fauna in their barcode availability. Fish barcode availability rates were much lower for threatening-to-invade taxa (~70%) than either native or introduced taxa (>95%; Fig. 3). This low rate is driven entirely by a recent assessment of Ponto-Caspian fishes^25^ that used physiological tolerances and ecological preferences to screen for species that might thrive in the Great Lakes were they to be introduced. All fishes added to the threatening-to-invade list based on assessments that screened for presence elsewhere in North America^26^,^27^ or invasiveness elsewhere in the world^28^,^29^ did have barcodes available. The one anuran on the threatening-to-invade list had barcodes (Table 2, Fig. 3). All invertebrates on the threatening-to-invade list had barcodes (Table 2), which is in distinct contrast to the situation for native or already-introduced invertebrates (Fig. 3).

An interesting counterpoint to the generally low barcoding rate for invertebrates compared to vertebrates is that a few invertebrate species are exceptionally well-represented. The Great Lakes species with the most barcodes in BOLD was a mosquito (*Aedes vexans*, ~2000 barcodes), and 8 other benthic macroinvertebrates had >300 barcodes (6 insects, 2 amphipods; all native). The zooplankton with the most barcodes was an introduced copepod (*Eurytemora affinis*, >300) and 6 native zooplankton had >100 barcodes (1 rotifer, 2 copepods, 3 branchiopods). The fish species with the most barcodes was the golden shiner (*Notemigonus crysoleucas*, >400) and 9 other fish species had >200 barcodes (2 native cyprinids, 2 introduced and one native salmonids, 3 native percids, 1 threatening-to-invade cyprinid). The most barcodes in BOLD for a Great Lakes amphibian was 95 (Fowler’s toad; *Anaxyrus fowleri*) and for a reptile was only 17 (garter snake; *Thamnophis sirtalis*).

Not all species lacking barcodes were missing from BOLD altogether. Finding a record in BOLD suggests that sequencing work on the species is underway (since creating such a record is a necessary precursor to using BOLD for assembling and managing DNA sequence data); in contrast, being missing from BOLD suggests a lack of attention to DNA sequencing for a species. The reptiles and amphibians lacking barcodes all had records in BOLD whereas the one extant fish lacking barcodes was missing (Table 1). Most threatening-to-invade fishes lacking barcodes were missing from BOLD entirely (Table 2). Among invertebrates, it was common for zooplankton, mollusks, and insects that lacked barcodes to have BOLD entries, whereas all leeches, oligochaetes, mites, and benthic crustaceans lacking barcodes were missing from BOLD entirely (Table 1). A few taxa had barcodes in BOLD but associated only with genus-level identities (noted in Supplementary Table S2).

The extant salamander and fish species lacking barcodes all had congener species with barcodes within the Great Lakes, whereas the one anuran species lacking barcodes had no congener. Sixty-seven percent of extant zooplankton and 73% of extant benthos species lacking barcodes had a congener with barcodes, and a barcoded congener from the Great Lakes was present for 56% and 53% of them, respectively. The 28 threatening-to-invade fish species lacking barcodes came from 14 genera; of which 2 had Great Lakes relatives with barcodes in BOLD (genera *Alosa* and *Neogobius*) and the other 12 are genera not currently found in the Great Lakes.

## Discussion

The ability to attach taxonomic labels to DNA sequences recovered from mixed-organism samples depends on the availability of comprehensive barcode reference libraries. Our study, which assessed the match between Great Lakes aquatic metazoan listings and catalogued COI mitochondrial DNA barcode sequences, has substantial implications concerning the current capacity to conduct aquatic bioassessment and invasive species monitoring using DNA-based identification. Notable findings are that comprehensive species inventories are needed and nontrivial to generate, that many extant invertebrate species currently lack cataloged barcodes, and that numerous extant aquatic invertebrates lack even the species-level resolution necessary to examine barcode matches. The level of barcode representation is poorest precisely for those organisms that are taxonomically least resolved and morphologically most challenging to identify. Threatening-to-invade species mostly have barcodes but these lists appear to be taxonomically incomplete. We expand on these topics and the current capacity for metabarcoding for bioassessment and aquatic invasive species monitoring below.

While the number of publications addressing the ability of DNA barcoding to assign species identities continues to expand (e.g., refs 30, 31, 32, 33, 34)our study is the first we are aware of that completes such an evaluation for an entire regional fauna. Most published studies focus on whether genetic differentiation patterns in the barcode locus allow species-level resolution rather than focusing (as we do) on whether reference barcodes are sufficiently available in the supporting databases. An exception is Kvist (ref. 35), who recently completed an analysis that compared, on a phylum by phylum basis, the world’s currently recognized invertebrate species (>1 million) against the number for which barcodes were available. Great Lakes fauna have substantially higher barcode availability rates in BOLD (Table 1) than Kvist’s worldwide averages – which were only 12% for arthropods, 11% for annelids, 10% for rotifers, and 6% for mollusks^35^.

Assembling species lists for Great Lakes metazoans and querying their barcode availability is a substantial task because of the number of organisms involved, the many data sources across which this information is spread, and constantly evolving taxonomic nomenclature. The Great Lakes basin spans broad latitudinal and environmental gradients and multiple distinct types of aquatic ecosystems. Biological communities differ across these gradients and subsystems, and biotic inventories are scattered across research and management entities and publication outlets (including peer-reviewed literature, agency reports, websites) with each source offering some unique taxa. Nomenclature varies across data sources, the nomenclature used by BOLD is not always current (such cases are noted in Supplementary Table S2), and some taxa have entries in BOLD under both current and previously recognized names. Such inconsistencies can be recognized and resolved – for example by searching multiple name variants, and beginning BOLD searches with a genus-level entry so as to return all species names under which sequences have been submitted – but require diligence on the part of researchers. There is no en-masse way of querying BOLD regarding which taxa from a list have barcodes; names have to be looked up individually, which is time-consuming for a large inventory.

The continuing interest in broad-scale bioassessment and non-native species monitoring raises the need to identify taxa from throughout the basin. For example, aquatic invasive species monitoring is often focused on port cities^36^ which have multiple human-mediated transport vectors (e.g., commercial shipping, recreational boating, aquarium dumping, bait release) and tend to be at ecotones (e.g., river-lake confluences) where natural processes bring a diversity of taxa together^37^. Evaluation of invasive species monitoring designs is best done using complete biological composition information, because encounter rates for rare species (whether native or not) are key to establishing sampling efficiency and detection probability^38^. Comprehensive species inventories and regionally appropriate identification keys covering a variety of life stages remain a research need that DNA-based identification can supplement but not entirely supplant, because existing taxonomic and biogeographic knowledge remains the basis for verification of barcode identities.

A substantial percentage of Great Lakes aquatic invertebrate species presently lack a barcode in the BOLD database. The percentage is particularly high among smaller zooplankton (ostracods, rotifers) and among non-insect benthic macroinvertebrates (crustaceans, mites, mollusks, annelids, etc.) – organisms for which morphological identification is also troublesome for reasons including small size, necessity for labor-intensive processing (e.g., slide mounts), lack of taxonomic keys and knowledge, lack of external differentiating characteristics (particular among immature life stages), and tendency for specimens to be damaged during collection. DNA sequencing and subsequent bioinformatics data processing still produce unique operational taxonomic units (OTUs) for organisms lacking barcodes, but assigning a species label is not possible. Over half of the species lacking barcodes had a barcoded congener such that an OTU could be assigned to a genus, but the remainder of species lacking barcodes could at best be assigned to family or order – a clear loss of biodiversity information even though richness can be assessed from the OTUs alone.

There is much work to be done developing barcode libraries before molecular taxonomy can provide complete species-level identification for mixed invertebrate samples^39^. In contrast, barcode libraries are already adequate to support DNA-based identification of vertebrate life stages where morphological identification is challenging. Collections of amphibian eggs and fish larvae – easily obtained in the field but difficult to identify in the laboratory – are viable monitoring targets with metabarcoding as the tool. But even among vertebrates, some of the taxa most challenging to identify morphologically are also those for which supporting barcodes are sparse (because researchers are reluctant to catalogue a barcode for a specimen of uncertain identity) or for which insufficient divergence in the barcode locus prevents distinguishing them genetically. For example, the genetic and ecological distinctness of *Coregonus* fishes are still actively being investigated^40^,^41^,^42^. Three of the seven *Coregonus* species included here had less than five barcodes in BOLD (Supplementary Table S2) while *C. reighardi* (which is possibly extirpated) lacked barcodes entirely. Some species in the *Cottus* genus of fishes are also hard to distinguish morphologically and genetically^10^,^41^; because there are several native Great Lakes *Cottus* as well as a *Cottus* on the threatening-to-invade list their confusion could potentially result in a non-native species not being recognized as such.

Based on our findings for threatening-to-invade species (Table 2), one could conclude that barcode availability is already adequate to support monitoring for them. All threatening-to-invade invertebrates had barcodes. Several threatening-to-invade Ponto-Caspian fishes did not have barcodes, but these were all from genera not currently found in the Great Lakes, meaning the likelihood of confusing their DNA sequences with extant species is small. However, the ability to detect new non-native species with DNA technology is probably not as good as threatening-to-invade-list results suggest, because the list appears to be taxonomically incomplete. For example the absence of clams and oligochaete worms from the threatening-to-invade list (Table 2) is conspicuous given that a high percentage of invertebrates already introduced to the Great Lakes come from these taxa.

A substantial percentage of invertebrates reported from Great Lakes waters are not resolved to species – even when the best taxonomic resolution across all inventories is used – and we expect the situation is similar in other waterbody types and regions. The percentage is likely to be higher for any single study, because specimen condition or life stage prevents species-level identification or resources and taxonomic expertise are lacking. The difficulty in identifying certain taxa to species and locating supporting keys and biogeographic information is not new to taxonomists, but does have implications for how DNA technology can advance biological understanding. Knowledge concerning aquatic metazoan biodiversity will clearly benefit from the capacity of DNA technology to assign species-level IDs to previously more poorly resolved taxonomic units. However, DNA technology can also raise new questions concerning biodiversity. For example, when DNA sequences are matched to barcodes for organisms not previously resolved to species, information to establish whether the species is native or introduced may be lacking. For groups with very poor taxonomic and biogeographic information, it may even be difficult to determine if the sequence is plausible versus sample contamination or DNA sequence “noise”. Some organism groups may turn out to have fairly complete barcode libraries despite poor taxonomic resolution from aquatic samples (e.g., flying insects are readily identifiable even if their aquatic larvae are difficult), but other understudied or difficult taxa are also poorly represented in barcode libraries (e.g., mites, ostracods, rotifers).

Attaining the capability for DNA-based identification to support biomonitoring in the Great Lakes and elsewhere requires a concerted effort to develop barcode libraries and physical voucher collections. Attention needs to be given to macroinvertebrates broadly and to certain taxonomically difficult vertebrates. The International Barcode of Life already has developed several successful campaigns to complete barcode databases for particular groups (e.g. Trichoptera Barcode of Life) or habitats (e.g. Polar Barcode of Life) that could be a model for further efforts. The infrastructure to build and share DNA databases on-line is already in place, and researchers from across the globe have the ability to contribute to and benefit from the continual expansion of barcode libraries. Our research group has initiated a practice of ‘library building’ as part of ongoing research into aquatic invasive species early detection strategies, whereby representative organisms from new taxa encountered (whether native or not) are set aside for DNA sequencing. We call upon the research community in the Great Lakes and elsewhere to do the same.

## Methods

We drew on a number of sources in order to compile reasonably complete lists of fishes, reptiles, amphibians, benthic macroinvertebrates, and zooplankton currently found in Great Lakes waters (extant lists), or considered likely to be introduced in the near future (threatening-to-invade lists). Extant species were categorized as non-native if included in lists of taxa originating from outside the Great Lakes basin^16^,^43^,^44^, otherwise they were assumed to be native (we ignored range expansion within the Great Lakes). We used ITIS (Integrated Taxonomic Information System; < www.itis.gov>) to check and update taxonomic nomenclature, and to generate the taxonomic hierarchy for each organism (i.e., phylum, class, order, family). There were quite a few taxa that were not resolved to species level in any report from Great Lakes waters, in which case we retained the most resolved taxonomic level that was reported (typically genus, sometimes family). Extant reptile and amphibian species were compiled from refs 45, 46, 47. Extant fishes were compiled from refs 48 and 49. Extant zooplankton were compiled from refs 43 and 50, 51, 52, 53, 54, 55, 56, 57, 58, 59, 60, 61, and taxa lists underlying ref. 62. Extant benthic macroinvertebrates were compiled from refs 43,56,57,59,60,63, 64, 65, 66, 67, 68, 69, 70, 71, 72, 73, 74 and lists provided by U.S. EPA’s Great Lakes National Program Office. Threatening-to-invade lists for all the target taxonomic groups were compiled from refs 25, 26, 27, 28, 29,75 and 76.

All taxa on the extant and threatening-to-invade lists that were resolved to species were queried in the BOLD database for the availability of barcodes (meaning sequences meeting BOLD’s criteria for being a barcode, i.e., at least 500 base-pairs long, with <1% ambiguous bases and detailed supporting information including electropherogram trace files). We first determined whether the species had a record in BOLD, and if so, recorded the number of barcodes present. Species that had records in BOLD but lacked barcodes were recorded as “zero barcodes” whereas species that lacked records entirely were recorded as “missing” – a distinction that helps discern between species for which DNA sequence work may be in progress versus species not currently receiving sequencing attention. For species that had no barcodes in BOLD or were missing from BOLD entirely, we recorded the nearest taxonomic level at which a relative with a barcode was found (e.g., same genus) and whether there was a Great Lakes congener with a barcode. Before concluding that a species lacked records in BOLD we also searched synonyms and older name variants.

Data analysis focused on summarizing the patterns of barcode availability by taxonomic group and native versus introduced status for taxa with species-level identification. We also summarized the frequency of taxa not being resolved to species. Because the BOLD catalogue is constantly expanding, the current number of barcodes for a given species is less informative than the broad patterns among species in barcode representation. A large number of barcodes is not necessary for confident DNA-based identification, but there should be enough sequences that within-species variability can be examined. To broadly capture the range in barcode availability without focusing on exact numbers, we summarized using three categories <5 barcodes (limited capability to examine variability), 5–25 barcodes (moderate capability to examine variability), and >25 barcodes (good capability to examine variability). Our BOLD searches spanned a period of roughly 6 months (late 2013 to early 2014), during which time the actual number of barcodes increased for some species but the barcode availability categories were stable.

We did not attempt to ascertain the taxonomic validity of the records in BOLD, nor examine the actual reference sequences. Part of the quality assurance of matching one’s own sequences to ones in BOLD would ordinarily include inspecting the source of the closely matching barcodes, but this goes well beyond our focus here of simply elucidating patterns of barcode availability. Sequences in BOLD are a mixture of “public” and “private” with the latter not being directly available for examination; private sequences are included among those queried when a test sequence is submitted for identification but BOLD only displays the percent match and taxonomic label, not the sequences themselves. In order to return private as well as public sequence availability, we did our searches using BOLD’s “search taxonomy” feature rather than the “public data portal”.

We did our searches in BOLD rather than the GenBank database because BOLD screens sequences with the specific goal of yielding reference barcodes attached to vouchered specimens and has a search interface well-suited to the task of querying COI barcode availability. We recognize that GenBank might yield barcodes for some species not found in BOLD as their COI sequence coverage is slightly different; however GenBank does not appear to differ appreciably from BOLD in the distribution of barcodes among taxonomic groups^35^. BOLD has somewhat better COI barcode coverage than GenBank for the 4 invertebrate phyla that numerically dominate our species inventory (e.g., Annelida 10.9% in BOLD vs. 10.6% in GenBank, Arthropoda 12.1 vs. 5.6%, Mollusca 6.1 vs. 5.8%, and Rotifera 10.0 vs. 4.4%; ref. 35), but the converse is true for other phyla.

## Additional Information

**How to cite this article**: Trebitz, A. S. *et al.* Potential for DNA-based identification of Great Lakes fauna: match and mismatch between taxa inventories and DNA barcode libraries. *Sci. Rep.* **5**, 12162; doi: 10.1038/srep12162 (2015).

## Supplementary Material

Supplementary Information

## Figures and Tables

**Table 1:** Summary of extant Great Lakes aquatic fauna and associated availability of COI mitochondrial DNA barcodes in the BOLD database.

Group	Genus ID only	Family ID only	Number of families|genera|species	Species in BOLD	Barcode category distribution (%) zero|<5|5–25|>25
*Vertebrates*					
Fishes	–	–	28|80|181	99%	–|4|41|55
Anurans	–	–	3|5|15	100%	6|27|40|27
Salamanders	–	–	6|11|20	100%	10|25|45|20
Snakes	–	–	2|7|12	100%	–|50|50|–
Turtles	–	–	4|9|10	100%	–|40|60|–
*Invertebrates*					
Branchiopods	1%	–	11|45|98	73%	8|28|53|11
Copepods	2%	–	12|35|59	47%	11|39|29|21
Ostracods	13%	–	3|6|7	43%	–|33|33|33
Rotifers	2%	–	22|53|201	36%	20|35|39|5
Leeches	–	–	4|12|18	56%	–|90|10|–
Oligochaetes	3%	2%	10|50|112	30%	–|82|9|9
Mites	41%	–	16|20|16	0%	n/a
Benthic crustacea^1^	–	–	8|15|36	61%	–|41|32|27
Coleopterans	44%	1%	16|68|52	92%	46|21|27|6
Odonates	–	–	10|52|189	79%	7|26|50|18
Dipterans	30%	3%	17|116|115	56%	3|29|46|22
EPT taxa^2^	15%	–	34|97|149	91%	2|16|38|44
Hemipterans	25%	2%	13|29|34	88%	28|48|21|3
Other insects^3^	55%	9%	8|12|4	50%	–|50|50|–
Clams & mussels	–	–	4|30|81	64%	13|31|46|10
Gastropods	–	–	10|35|67	43%	3|45|41|10
Other benthos^4^	26%	–	27|31|26	35%	–|78|22|–

Taxa are broken out to finer categories here than in the graphs. Because of non-species level IDs, number of genera equals or exceeds number of species for some groups.

^1^Orders Amphipoda, Decapoda, Isopoda, Mysida.

^2^Orders Ephemeroptera, Plecoptera, and Trichoptera.

^3^Orders Collembola, Lepidoptera, Megaloptera, Neuroptera.

^4^Phyla Bryozoa, Cnidaria, Kamptozoa, Nematomorpha, Nemertea, Platyhelminthes, Porifera, and Tardigrada.

**Table 2:** Summary of Great Lakes aquatic fauna threatening-to-invade list and associated availability of COI mitochondrial DNA barcodes in the BOLD database.

Group	Number of families|genera|species	Species in BOLD	Barcode category distribution (%) zero|<5|5–25|>25
*Vertebrates*			
Fishes	27|64|97	73%	3|21|41|35
Anurans	1|1|1	100%	–|100|–|–
*Invertebrates*			
Copepods	1|1|1	100%	–|100|–|–
Benthic Crustacea^1^	6|8|10	100%	–|50|30|20
Gastropods	4|4|4	100%	–|25|25|50
Other benthos^2^	3|3|3	100%	–|67|33|–

All threatening-to-invade taxa are resolved to species.

^1^Orders Amphipoda, Decapoda, Isopoda, Mysida – same orders as in Table 1.

^2^Orders Rhizostomeae, Opisthorchiida, Strigeidida – not same orders as in Table 1.
