# Supplementary material for: Potential for DNA-based identification of Great Lakes fauna: match and mismatch between taxa inventories and DNA barcode libraries
Source: Sci Rep. 2015 Jul 22;5:12162. doi: 10.1038/srep12162 (PMC4510495; doi:10.1038/srep12162)
Supplement: Supplementary Information [file srep12162-s1.doc]

**Potential for DNA-based identification of Great Lakes fauna: match and mismatch between taxa inventories and DNA barcode libraries**

Anett S. Trebitz*, Joel C. Hoffman, George W. Grant, Tyler M. Billehus, and Erik M. Pilgrim

**Supplementary Table S1.** Compiled list of extant Great Lakes aquatic metazoans for which best reported taxonomic resolution is not to species. Nomenclature follows current ITIS listing. The one introduced taxon has its name offset with asterisks.

| **Class** | **Order** | **Family** | **Genus** | **ID level** |
| --- | --- | --- | --- | --- |
| **Phylum *Annelida*** | | | | |
| Oligochaeta | Branchiobdellida | Branchiobdellidae |  | Family |
| Oligochaeta | Haplotaxida | Enchytraeidae | *Enchytraeus* | Genus |
| Oligochaeta | Haplotaxida | Enchytraeidae | *Mesenchytraeus* | Genus |
| Oligochaeta | Haplotaxida | Tubificidae | *Teneridrilus* | Genus |
| Oligochaeta | Haplotaxida | Tubificidae | *Thalassodrilus* | Genus |
| Sedentaria | Canalipalpata | Serpulidae |  | Family |
| ***Phylum Arthropoda - Chelicerata*** | | | | |
| Arachnida | Sarcoptiformes | Cymbaeremaeidae | *Scapheremaeus* | Genus |
| Arachnida | Sarcoptiformes | Damaeidae | *Belba* | Genus |
| Arachnida | Sarcoptiformes | Hydrozetidae | *Hydrozetes* | Genus |
| Arachnida | Sarcoptiformes | Malaconothridae | *Trimalaconothrus* | Genus |
| Arachnida | Trombidiformes | Hydrachnidae | *Hydrachna* | Genus |
| Arachnida | Trombidiformes | Hygrobatidae | *Atractides* | Genus |
| Arachnida | Trombidiformes | Hygrobatidae | *Hygrobates* | Genus |
| Arachnida | Trombidiformes | Lebertiidae | *Lebertia* | Genus |
| Arachnida | Trombidiformes | Mideopsidae | *Mideopsis* | Genus |
| Arachnida | Trombidiformes | Torrenticolidae | *Torrenticola* | Genus |
| Arachnida | Trombidiformes | Unionicolidae | *Unionicola* | Genus |
| ***Phylum Arthropoda - Crustacea*** | | | | |
| Branchiopoda | Diplostraca | Daphniidae | *Megafenestra* | Genus |
| Maxillopoda | Harpacticoida | Canthocamptidae | *Maraenobiotus* | Genus |
| Ostracoda | Podocopida | Cyprididae | *Pelocypris* | Genus |
| ***Phylum Arthropoda – Hexapoda*** | | | | |
| Entognatha | Collembola | Istotomidae | *Isotomurus* | Genus |
| Insecta | Coleoptera | Chrysomelidae | *Donacia* | Genus |
| Insecta | Coleoptera | Chrysomelidae | *Pyrrhalta* | Genus |
| Insecta | Coleoptera | Curculionidae | *Auleutes* | Genus |
| Insecta | Coleoptera | Curculionidae | *Lixus* | Genus |
| Insecta | Coleoptera | Curculionidae | *Pelonomus* | Genus |
| Insecta | Coleoptera | Curculionidae | *Rhinoncus* | Genus |
| Insecta | Coleoptera | Curculionidae | *Tanysphyrus* | Genus |
| Insecta | Coleoptera | Dryopidae | *Helichus* | Genus |
| Insecta | Coleoptera | Dytiscidae | *Agabetes* | Genus |
| Insecta | Coleoptera | Dytiscidae | *Agabus* | Genus |
| Insecta | Coleoptera | Dytiscidae | *Bidessus* | Genus |
| Insecta | Coleoptera | Dytiscidae | *Celina* | Genus |
| Insecta | Coleoptera | Dytiscidae | *Copelatus* | Genus |
| Insecta | Coleoptera | Dytiscidae | *Coptotomus* | Genus |
| Insecta | Coleoptera | Dytiscidae | *Cybister* | Genus |
| Insecta | Coleoptera | Dytiscidae | *Graphoderus* | Genus |
| Insecta | Coleoptera | Dytiscidae | *Hydaticus* | Genus |
| Insecta | Coleoptera | Dytiscidae | *Hydrovatus* | Genus |
| Insecta | Coleoptera | Dytiscidae | *Ilybius* | Genus |
| Insecta | Coleoptera | Dytiscidae | *Laccornis* | Genus |
| Insecta | Coleoptera | Dytiscidae | *Liodessus* | Genus |
| Insecta | Coleoptera | Dytiscidae | *Matus* | Genus |
| Insecta | Coleoptera | Dytiscidae | *Neoporus* | Genus |
| Insecta | Coleoptera | Dytiscidae | *Oreodytes* | Genus |
| Insecta | Coleoptera | Dytiscidae | *Rhantus* | Genus |
| Insecta | Coleoptera | Dytiscidae | *Uvarus* | Genus |
| Insecta | Coleoptera | Elmidae | *Microcylloepus* | Genus |
| Insecta | Coleoptera | Elmidae | *Promoresia* | Genus |
| Insecta | Coleoptera | Gyrinidae | *Dineutus* | Genus |
| Insecta | Coleoptera | Helophoridae | *Helophorus* | Genus |
| Insecta | Coleoptera | Hydrochidae | *Hydrochus* | Genus |
| Insecta | Coleoptera | Hydrochidae | *Crenitis* | Genus |
| Insecta | Coleoptera | Hydrophilidae | *Cymbiodyta* | Genus |
| Insecta | Coleoptera | Hydrophilidae | *Helochares* | Genus |
| Insecta | Coleoptera | Hydrophilidae | *Helocombus* | Genus |
| Insecta | Coleoptera | Hydrophilidae | *Hydrophilus* | Genus |
| Insecta | Coleoptera | Hydrophilidae | *Laccobius* | Genus |
| Insecta | Coleoptera | Hydrophilidae | *Tropisternus* | Genus |
| Insecta | Coleoptera | Noteridae | *Pronoterus* | Genus |
| Insecta | Coleoptera | Sciritidae | *Cyphon* | Genus |
| Insecta | Coleoptera | Staphylinidae |  | Family |
| Insecta | Diptera | Ceratopogonidae | *Atrichopogon* | Genus |
| Insecta | Diptera | Ceratopogonidae | *Bezzia* | Genus |
| Insecta | Diptera | Ceratopogonidae | *Culicoides* | Genus |
| Insecta | Diptera | Ceratopogonidae | *Probezzia* | Genus |
| Insecta | Diptera | Chaoboridae | *Mochlonyx* | Genus |
| Insecta | Diptera | Chironomidae | *Acalcarella* | Genus |
| Insecta | Diptera | Chironomidae | *Acricotopus* | Genus |
| Insecta | Diptera | Chironomidae | *Axarus* | Genus |
| Insecta | Diptera | Chironomidae | *Chaetocladius* | Genus |
| Insecta | Diptera | Chironomidae | *Cladopelma* | Genus |
| Insecta | Diptera | Chironomidae | *Clinotanypus* | Genus |
| Insecta | Diptera | Chironomidae | *Conchapelopia* | Genus |
| Insecta | Diptera | Chironomidae | *Cryptotendipes* | Genus |
| Insecta | Diptera | Chironomidae | *Demeijerea* | Genus |
| Insecta | Diptera | Chironomidae | *Demicryptochironomus* | Genus |
| Insecta | Diptera | Chironomidae | *Diamesa* | Genus |
| Insecta | Diptera | Chironomidae | *Doncricotopus* | Genus |
| Insecta | Diptera | Chironomidae | *Eukiefferiella* | Genus |
| Insecta | Diptera | Chironomidae | *Kloosia*  (not found in ITIS) | Genus |
| Insecta | Diptera | Chironomidae | *Larsia* | Genus |
| Insecta | Diptera | Chironomidae | *Limnophyes* | Genus |
| Insecta | Diptera | Chironomidae | *Lipinella* | Genus |
| Insecta | Diptera | Chironomidae | *Nanocladius* | Genus |
| Insecta | Diptera | Chironomidae | *Natarsia* | Genus |
| Insecta | Diptera | Chironomidae | *Nilothauma* | Genus |
| Insecta | Diptera | Chironomidae | *Paracricotopus* | Genus |
| Insecta | Diptera | Chironomidae | *Paramerina* | Genus |
| Insecta | Diptera | Chironomidae | *Paraphaenocladius* | Genus |
| Insecta | Diptera | Chironomidae | *Paratanytarsus* | Genus |
| Insecta | Diptera | Chironomidae | *Paratendipes* | Genus |
| Insecta | Diptera | Chironomidae | *Pentaneura* | Genus |
| Insecta | Diptera | Chironomidae | *Phaenopsectra* | Genus |
| Insecta | Diptera | Chironomidae | *Pseudosmittia* | Genus |
| Insecta | Diptera | Chironomidae | *Rheocricotopus* | Genus |
| Insecta | Diptera | Chironomidae | *Rheosmittia* | Genus |
| Insecta | Diptera | Chironomidae | *Sergentia* | Genus |
| Insecta | Diptera | Chironomidae | *Smittia* | Genus |
| Insecta | Diptera | Chironomidae | *Stictochironomus* | Genus |
| Insecta | Diptera | Chironomidae | *Stilocladius* | Genus |
| Insecta | Diptera | Chironomidae | *Sympotthastia* | Genus |
| Insecta | Diptera | Chironomidae | *Thienemanniella* | Genus |
| Insecta | Diptera | Chironomidae | *Thienemanniola* | Genus |
| Insecta | Diptera | Chironomidae | *Zalutschia* | Genus |
| Insecta | Diptera | Chironomidae | *Zavrelimyia* | Genus |
| Insecta | Diptera | Culicidae | *Anopheles* | Genus |
| Insecta | Diptera | Culicidae | *Mansonia* | Genus |
| Insecta | Diptera | Dixidae | *Dixella* | Genus |
| Insecta | Diptera | Dolichopodidae |  | Family |
| Insecta | Diptera | Empididae | *Hemerodromia* | Genus |
| Insecta | Diptera | Ephydridae |  | Family |
| Insecta | Diptera | Mycetophilidae |  | Family |
| Insecta | Diptera | Pelecorhynchidae | *Glutops* | Genus |
| Insecta | Diptera | Psychodidae | *Pericoma* | Genus |
| Insecta | Diptera | Psychodidae | *Psychoda* | Genus |
| Insecta | Diptera | Sciaridae |  | Family |
| Insecta | Diptera | Sciomyzidae | *Sepedon* | Genus |
| Insecta | Diptera | Stratiomyidae | *Myxosargus* | Genus |
| Insecta | Diptera | Stratiomyidae | *Odontomyia* | Genus |
| Insecta | Diptera | Stratiomyidae | *Stratiomys* | Genus |
| Insecta | Diptera | Syrphidae |  | Family |
| Insecta | Diptera | Tabanidae |  | Family |
| Insecta | Diptera | Tipulidae | *Antocha* | Genus |
| Insecta | Diptera | Tipulidae | *Tipula* | Genus |
| Insecta | Ephemeroptera | Ameletidae | *Ameletus* | Genus |
| Insecta | Ephemeroptera | Baetidae | *Baetis* | Genus |
| Insecta | Ephemeroptera | Baetidae | *Callibaetis* | Genus |
| Insecta | Ephemeroptera | Baetidae | *Centroptilum* | Genus |
| Insecta | Ephemeroptera | Baetidae | *Paracloeodes* | Genus |
| Insecta | Ephemeroptera | Baetidae | *Procloeon* | Genus |
| Insecta | Ephemeroptera | Baetidae | *Pseudocloeon* | Genus |
| Insecta | Ephemeroptera | Caenidae | *Brachycercus* | Genus |
| Insecta | Ephemeroptera | Caenidae | *Cloeon* | Genus |
| Insecta | Ephemeroptera | Heptageniidae | *Anepeorus* | Genus |
| Insecta | Ephemeroptera | Leptohyphidae | *Tricorythodes* | Genus |
| Insecta | Ephemeroptera | Leptophlebiidae | *Leptophlebia* | Genus |
| Insecta | Ephemeroptera | Siphlonuridae | *Siphlonurus* | Genus |
| Insecta | Hemiptera | Corixidae | *Dasycorixa* | Genus |
| Insecta | Hemiptera | Gerridae | *Gerris* | Genus |
| Insecta | Hemiptera | Gerridae | *Rheumatobates* | Genus |
| Insecta | Hemiptera | Gerridae | *Trepobates* | Genus |
| Insecta | Hemiptera | Hebridae | *Merragata* | Genus |
| Insecta | Hemiptera | Naucoridae | *Pelocoris* | Genus |
| Insecta | Hemiptera | Nepidae | *Nepa* | Genus |
| Insecta | Hemiptera | Notonectidae | *Buenoa* | Genus |
| Insecta | Hemiptera | Oligoneuriidae |  | Family |
| Insecta | Hemiptera | Saldidae | *Micracanthia* | Genus |
| Insecta | Hemiptera | Veliidae | *Microvelia* | Genus |
| Insecta | Hemiptera | Veliidae | *Paravelia* | Genus |
| Insecta | Hemiptera | Veliidae | *Rhagovelia* | Genus |
| Insecta | Lepidoptera | Braconidae |  | Family |
| Insecta | Lepidoptera | Crambidae | *Nymphula* | Genus |
| Insecta | Lepidoptera | Crambidae | *Parapoynx* | Genus |
| Insecta | Lepidoptera | Crambidae | *Petrophila* | Genus |
| Insecta | Lepidoptera | Noctuidae | *Archanara* | Genus |
| Insecta | Lepidoptera | Noctuidae | *Bellura* | Genus |
| Insecta | Megaloptera | Corydalidae | *Chauliodes* | Genus |
| Insecta | Megaloptera | Sialidae | *Sialis* | Genus |
| Insecta | Plecoptera | Chloroperlidae | *Alloperla* | Genus |
| Insecta | Trichoptera | Apataniidae | *Apatania* | Genus |
| Insecta | Trichoptera | Brachycentridae | *Micrasema* | Genus |
| Insecta | Trichoptera | Glossosomatidae | *Glossosoma* | Genus |
| Insecta | Trichoptera | Hydropsychidae | *Symphitopsyche* | Genus |
| Insecta | Trichoptera | Leptoceridae | *Ylodes* | Genus |
| Insecta | Trichoptera | Limnephilidae | *Anabolia* | Genus |
| Insecta | Trichoptera | Limnephilidae | *Dicosmoecus* | Genus |
| Insecta | Trichoptera | Limnephilidae | *Goerinae* | Genus |
| Insecta | Trichoptera | Limnephilidae | *Grammotaulius* | Genus |
| Insecta | Trichoptera | Limnephilidae | *Lenarchus* | Genus |
| Insecta | Trichoptera | Limnephilidae | *Pycnopsyche* | Genus |
| Insecta | Trichoptera | Phryganeidae | *Ptilostomis* | Genus |
| ***Phylum Gastrotricha*** | | | | |
| n/a | Chaetonotida | Chaetonotidae | *Chaetonotus* | Genus |
| ***Phylum Mollusca*** |  |  |  |  |
| Gastropoda | Neotaenioglossa | Hydrobiidae | *Birgella* | Genus |
| ***Phylum Nematoda*** | | | | |
| Chromadorea | Tylenchida | Criconematidae | *Criconemoides* | Genus |
| Dorylaimea | Dorylaimida | Dorylaimidae | *Dorylaimus* | Genus |
| Enoplea | Enoplida | Tripylidae | *Tobrilus* | Genus |
| ***Phylum Platyhelminthes*** | | | | |
| n/a | Catenulida | Stenostomidae | *Stenostomum* | Genus |
| Trematoda | Opisthorchiida | Acanthostomatidae | *** Timoniella*** | Genus |
| Trepaxonemata | Neoophora | Dalyelliidae | *Microdalyella* | Genus |
| Trepaxonemata | Neoophora | Typhloplanidae | *Mesostoma* | Genus |
| ***Phylum Rotifera*** | | | | |
| Bdelloidea | Philodinidae | Philodina | *Philodina* | Genus |
| Monogonta | Flosculariaceae | Flosculariidae | *Floscularia* | Genus |
| Monogonta | Flosculariaceae | Flosculariidae | *Sinantherina* | Genus |
| Monogonta | Ploima | Lepadellidae | *Colurella* | Genus |
| Monogonta | Ploima | Lepadellidae | *Lepadella* | Genus |
| ***Phylum Tardigrada*** | | | | |
| Eutardigrada | Parachela | Macrobiotidae | *Macrobiotus* | Genus |

**Supplementary Table S2**. Compiled list of extant Great Lakes aquatic metazoan taxa that are resolved to species. Final column gives their current status with respect to availability of barcodes in BOLD, with categories used being “lacking” (species missing from BOLD entirely), “genus” (barcodes labeled only to genus), “zero” (species listed in BOLD but with zero barcodes), “<5”, “5to25”, or “>25”. Nomenclature follows current ITIS listing, but cases where BOLD records were found under a different name are noted in parentheses. Introduced taxon have their name offset with asterisks.

| **Class** | **Order** | | **Family** | **Genus and species** | | **barcode status** |
| --- | --- | --- | --- | --- | --- | --- |
| **Phylum *Annelida*** |  | |  |  | |  |
| Hirudinea | Arhynchobdellida | | Erpobdellidae | Dina dubia | | lacking |
| Hirudinea | Arhynchobdellida | | Erpobdellidae | Dina parva | | lacking |
| Hirudinea | Arhynchobdellida | | Erpobdellidae | Erpobdella punctata | | <5 |
| Hirudinea | Arhynchobdellida | | Erpobdellidae | Nephelopsis obscura | | <5 |
| Hirudinea | Arhynchobdellida | | Haemopidae | Haemopis grandis | | <5 |
| Hirudinea | Rhynchobdellida | | Glossiphoniidae | Actinobdella pediculata | | lacking |
| Hirudinea | Rhynchobdellida | | Glossiphoniidae | Alboglossiphonia heteroclita | | lacking |
| Hirudinea | Rhynchobdellida | | Glossiphoniidae | Desserobdella phalera | | lacking |
| Hirudinea | Rhynchobdellida | | Glossiphoniidae | Gloiobdella elongata | | lacking |
| Hirudinea | Rhynchobdellida | | Glossiphoniidae | Glossiphonia complanata | | <5 |
| Hirudinea | Rhynchobdellida | | Glossiphoniidae | Helobdella elongata | | <5 |
| Hirudinea | Rhynchobdellida | | Glossiphoniidae | Helobdella fusca | | <5 |
| Hirudinea | Rhynchobdellida | | Glossiphoniidae | Helobdella stagnalis | | 5to25 |
| Hirudinea | Rhynchobdellida | | Glossiphoniidae | Helobdella triserialis | | <5 |
| Hirudinea | Rhynchobdellida | | Glossiphoniidae | Placobdella montifera | | <5 |
| Hirudinea | Rhynchobdellida | | Piscicolidae | Piscicola geometra | | <5 |
| Hirudinea | Rhynchobdellida | | Piscicolidae | Piscicola milneri | | lacking |
| Hirudinea | Rhynchobdellida | | Piscicolidae | Piscicola punctata | | lacking |
| Oligochaeta | Haplotaxida | | Enchytraeidae | Barbidrilus paucisetus | | lacking |
| Oligochaeta | Haplotaxida | | Haplotaxidae | Haplotaxis gordioides | | lacking |
| Oligochaeta | Haplotaxida | | Naididae | Allonais pectinata | | lacking |
| Oligochaeta | Haplotaxida | | Naididae | Amphichaeta americana | | lacking |
| Oligochaeta | Haplotaxida | | Naididae | Amphichaeta leydigi | | lacking |
| Oligochaeta | Haplotaxida | | Naididae | Arcteonais lomondi | | <5 |
| Oligochaeta | Haplotaxida | | Naididae | Bratislavia unidentata | | lacking |
| Oligochaeta | Haplotaxida | | Naididae | Chaetogaster diaphanus | | <5 |
| Oligochaeta | Haplotaxida | | Naididae | Chaetogaster diastrophus | | <5 |
| Oligochaeta | Haplotaxida | | Naididae | Chaetogaster limnaei | | <5 |
| Oligochaeta | Haplotaxida | | Naididae | Chaetogaster longi | | lacking |
| Oligochaeta | Haplotaxida | | Naididae | Chaetogaster setosus | | lacking |
| Oligochaeta | Haplotaxida | | Naididae | Dero digitata | | <5 |
| Oligochaeta | Haplotaxida | | Naididae | Dero flabelliger | | lacking |
| Oligochaeta | Haplotaxida | | Naididae | Dero furcata | | <5 |
| Oligochaeta | Haplotaxida | | Naididae | Dero nivea | | lacking |
| Oligochaeta | Haplotaxida | | Naididae | Dero obtusa | | <5 |
| Oligochaeta | Haplotaxida | | Naididae | Dero vaga | | <5 |
| Oligochaeta | Haplotaxida | | Naididae | Haemonais waldvogeli | | lacking |
| Oligochaeta | Haplotaxida | | Naididae | Nais alpina | | lacking |
| Oligochaeta | Haplotaxida | | Naididae | Nais barbata | | <5 |
| Oligochaeta | Haplotaxida | | Naididae | Nais behningi | | lacking |
| Oligochaeta | Haplotaxida | | Naididae | Nais bretscheri | | <5 |
| Oligochaeta | Haplotaxida | | Naididae | Nais communis | | <5 |
| Oligochaeta | Haplotaxida | | Naididae | Nais elinguis | | >25 |
| Oligochaeta | Haplotaxida | | Naididae | Nais pardalis | | lacking |
| Oligochaeta | Haplotaxida | | Naididae | Nais pseudobtusa | | lacking |
| Oligochaeta | Haplotaxida | | Naididae | Nais simplex | | lacking |
| Oligochaeta | Haplotaxida | | Naididae | Nais variabilis | | 5to25 |
| Oligochaeta | Haplotaxida | | Naididae | Ophidonais serpentina | | <5 |
| Oligochaeta | Haplotaxida | | Naididae | Paranais frici | | <5 |
| Oligochaeta | Haplotaxida | | Naididae | Paranais litoralis | | <5 |
| Oligochaeta | Haplotaxida | | Naididae | Piguetiella blanci | | lacking |
| Oligochaeta | Haplotaxida | | Naididae | Piguetiella michiganensis | | <5 |
| Oligochaeta | Haplotaxida | | Naididae | **Pristina acuminate** | | lacking |
| Oligochaeta | Haplotaxida | | Naididae | Pristina aequiseta | | <5 |
| Oligochaeta | Haplotaxida | | Naididae | Pristina breviseta | | lacking |
| Oligochaeta | Haplotaxida | | Naididae | Pristina foreli | | lacking |
| Oligochaeta | Haplotaxida | | Naididae | Pristina jenkinae | | lacking |
| Oligochaeta | Haplotaxida | | Naididae | Pristina leidyi | | <5 |
| Oligochaeta | Haplotaxida | | Naididae | Pristina longiseta | | <5 |
| Oligochaeta | Haplotaxida | | Naididae | Pristina longisoma | | lacking |
| Oligochaeta | Haplotaxida | | Naididae | Pristina osborni | | <5 |
| Oligochaeta | Haplotaxida | | Naididae | Pristina plumaseta | | lacking |
| Oligochaeta | Haplotaxida | | Naididae | Pristina sima | | lacking |
| Oligochaeta | Haplotaxida | | Naididae | Pristinella acuminata | | lacking |
| Oligochaeta | Haplotaxida | | Naididae | Pristinella osborni | | lacking |
| Oligochaeta | Haplotaxida | | Naididae | **Ripistes parasita** | | <5 |
| Oligochaeta | Haplotaxida | | Naididae | Slavina appendiculata | | <5 |
| Oligochaeta | Haplotaxida | | Naididae | Specaria josinae | | <5 |
| Oligochaeta | Haplotaxida | | Naididae | Stephensoniana trivandrana | | lacking |
| Oligochaeta | Haplotaxida | | Naididae | Stylaria fossularis | | <5 |
| Oligochaeta | Haplotaxida | | Naididae | Stylaria lacustris | | 5to25 |
| Oligochaeta | Haplotaxida | | Naididae | Uncinais uncinata | | lacking |
| Oligochaeta | Haplotaxida | | Naididae | Vejdovskyella comata | | <5 |
| Oligochaeta | Haplotaxida | | Naididae | Vejdovskyella intermedia | | lacking |
| Oligochaeta | Haplotaxida | | Sparganophilidae | Sparganophilus eiseni | | <5 |
| Oligochaeta | Haplotaxida | | Tubificidae | Aulodrilus americanus | | lacking |
| Oligochaeta | Haplotaxida | | Tubificidae | Aulodrilus limnobius | | lacking |
| Oligochaeta | Haplotaxida | | Tubificidae | Aulodrilus pigueti | | lacking |
| Oligochaeta | Haplotaxida | | Tubificidae | Aulodrilus pluriseta | | lacking |
| Oligochaeta | Haplotaxida | | Tubificidae | Bothrioneurum vejdovskyanum | | lacking |
| Oligochaeta | Haplotaxida | | Tubificidae | **Branchiura sowerbyi** | | <5 |
| Oligochaeta | Haplotaxida | | Tubificidae | **Gianius aquaedulcis** | | lacking |
| Oligochaeta | Haplotaxida | | Tubificidae | Ilyodrilus templetoni | | lacking |
| Oligochaeta | Haplotaxida | | Tubificidae | Isochaetides curvisetosus | | lacking |
| Oligochaeta | Haplotaxida | | Tubificidae | Isochaetides freyi | | lacking |
| Oligochaeta | Haplotaxida | | Tubificidae | Limnodrilus angustipenis | | lacking |
| Oligochaeta | Haplotaxida | | Tubificidae | Limnodrilus cervix | | lacking |
| Oligochaeta | Haplotaxida | | Tubificidae | Limnodrilus claparedianus | | lacking |
| Oligochaeta | Haplotaxida | | Tubificidae | Limnodrilus hoffmeisteri | | <5 |
| Oligochaeta | Haplotaxida | | Tubificidae | Limnodrilus maumeensis | | lacking |
| Oligochaeta | Haplotaxida | | Tubificidae | Limnodrilus profundicola | | lacking |
| Oligochaeta | Haplotaxida | | Tubificidae | Limnodrilus torilipenis | | lacking |
| Oligochaeta | Haplotaxida | | Tubificidae | Limnodrilus udekemianus | | lacking |
| Oligochaeta | Haplotaxida | | Tubificidae | Peloscolex ferox | | lacking |
| Oligochaeta | Haplotaxida | | Tubificidae | Peloscolex freyi | | lacking |
| Oligochaeta | Haplotaxida | | Tubificidae | Peloscolex multisetosus | | lacking |
| Oligochaeta | Haplotaxida | | Tubificidae | Peloscolex superiorensis | | lacking |
| Oligochaeta | Haplotaxida | | Tubificidae | Peloscolex variegatus | | lacking |
| Oligochaeta | Haplotaxida | | Tubificidae | Phallodrilus aquaedulcis | | lacking |
| Oligochaeta | Haplotaxida | | Tubificidae | Phallodrilus hallae | | lacking |
| Oligochaeta | Haplotaxida | | Tubificidae | Potamothrix bavaricus | | lacking |
| Oligochaeta | Haplotaxida | | Tubificidae | **Potamothrix bedoti** | | lacking |
| Oligochaeta | Haplotaxida | | Tubificidae | Potamothrix hammoniensis | | lacking |
| Oligochaeta | Haplotaxida | | Tubificidae | **Potamothrix moldaviensis** | | lacking |
| Oligochaeta | Haplotaxida | | Tubificidae | **Potamothrix vejdovskyi**F149 | | lacking |
| Oligochaeta | Haplotaxida | | Tubificidae | Psammoryctides barbatus | | lacking |
| Oligochaeta | Haplotaxida | | Tubificidae | Psammoryctides californianus | | lacking |
| Oligochaeta | Haplotaxida | | Tubificidae | Psammoryctides curvisetosus | | lacking |
| Oligochaeta | Haplotaxida | | Tubificidae | Quistradrilus multisetosus | | lacking |
| Oligochaeta | Haplotaxida | | Tubificidae | Rhyacodrilus coccineus | | lacking |
| Oligochaeta | Haplotaxida | | Tubificidae | Rhyacodrilus montanus | | lacking |
| Oligochaeta | Haplotaxida | | Tubificidae | Rhyacodrilus sodalis | | lacking |
| Oligochaeta | Haplotaxida | | Tubificidae | Spirosperma ferox | | lacking |
| Oligochaeta | Haplotaxida | | Tubificidae | Spirosperma nikolskyi | | lacking |
| Oligochaeta | Haplotaxida | | Tubificidae | Trasserkidrilus harmani | | lacking |
| Oligochaeta | Haplotaxida | | Tubificidae | Trasserkidrilus kessleri | | lacking |
| Oligochaeta | Haplotaxida | | Tubificidae | Trasserkidrilus superiorensis | | lacking |
| Oligochaeta | Haplotaxida | | Tubificidae | Tubifex ignotus | | lacking |
| Oligochaeta | Haplotaxida | | Tubificidae | Tubifex newaensis | | lacking |
| Oligochaeta | Haplotaxida | | Tubificidae | Tubifex tubifex | | >25 |
| Oligochaeta | Haplotaxida | | Tubificidae | Varichaetadrilus angustipenis | | lacking |
| Oligochaeta | Lumbriculida | | Lumbriculidae | Eclipidrilus lacustris | | lacking |
| Oligochaeta | Lumbriculida | | Lumbriculidae | Eiseniella tetraedra | | 5to25 |
| Oligochaeta | Lumbriculida | | Lumbriculidae | Lumbriculus variegatus | | >25 |
| Oligochaeta | Lumbriculida | | Lumbriculidae | Stylodrilus heringianus | | <5 |
| Oligochaeta | N/A | | Aeolosomatidae | Aeolosoma beddardi | | lacking |
| Oligochaeta | N/A | | Aeolosomatidae | Aeolosoma hemprichi | | lacking |
| Oligochaeta | N/A | | Aeolosomatidae | Aeolosoma leidyi | | lacking |
| Oligochaeta | N/A | | Aeolosomatidae | Aeolosoma tenebrarum | | lacking |
| Sedentaria | Canalipalpata | | Sabellidae | Manayunkia speciosa | | lacking |
| **Phylum** ***Arthropoda - Chelicerata*** |  | |  |  | |  |
| Arachnida | Mesostigmata | | Laelaptidae | Gammaridacarus brevisternalis | | lacking |
| Arachnida | Trombidiformes | | Aturidae | Aturus deceptor | | lacking |
| Arachnida | Trombidiformes | | Aturidae | Aturus desquamatus | | lacking |
| Arachnida | Trombidiformes | | Axonopsidae | Ljania bipapillata bipapillata | | lacking |
| Arachnida | Trombidiformes | | Axonopsidae | Ljania bipapillata pupurea | | lacking |
| Arachnida | Trombidiformes | | Limnesiidae | Limnesia cornuta | | lacking |
| Arachnida | Trombidiformes | | Mideopsidae | Forelia cayuga | | lacking |
| Arachnida | Trombidiformes | | Mideopsidae | Forelia onondaga | | lacking |
| Arachnida | Trombidiformes | | Mideopsidae | Piona rotunda | | lacking |
| Arachnida | Trombidiformes | | Sperchontidae | Sperchon mitchelli | | lacking |
| Arachnida | Trombidiformes | | Sperchontidae | Sperchonopsis ecphyma | | lacking |
| Arachnida | Trombidiformes | | Sperchontidae | Sperchonopsis ovalis | | lacking |
| Arachnida | Trombidiformes | | Sperchontidae | Sperchonopsis verrucosa | | lacking |
| Arachnida | Trombidiformes | | Teutoniidae | Teutonia lunata | | lacking |
| Arachnida | Trombidiformes | | Teutoniidae | Teutonia lundbladi | | lacking |
| Arachnida | Trombidiformes | | Teutoniidae | Teutonia setifera | | lacking |
| **Phylum *Arthropoda - Crustacea*** |  | |  |  | |  |
| Branchiopoda | Diplostraca | | Bosminidae | *Bosmina liederi* | | 5to25 |
| Branchiopoda | Diplostraca | | Bosminidae | *Bosmina longirostris* | | 5to25 |
| Branchiopoda | Diplostraca | | Bosminidae | ***Eubosmina coregoni***  (in BOLD as Bosmina coregoni) | | 5to25 |
| Branchiopoda | Diplostraca | | Bosminidae | *Eubosmina longispina* | | lacking |
| Branchiopoda | Diplostraca | | Bosminidae | ***Eubosmina maritima*** | | lacking |
| Branchiopoda | Diplostraca | | Cercopagididae | ***Bythotrephes longimanus*** | | 5to25 |
| Branchiopoda | Diplostraca | | Cercopagididae | ***Cercopagis pengoi*** | | 5to25 |
| Branchiopoda | Diplostraca | | Chydoridae | *Acroperus harpae* | | 5to25 |
| Branchiopoda | Diplostraca | | Chydoridae | *Alona affinis* | | 5to25 |
| Branchiopoda | Diplostraca | | Chydoridae | *Alona barbulata* | | lacking |
| Branchiopoda | Diplostraca | | Chydoridae | *Alona circumfimbriata* | | <5 |
| Branchiopoda | Diplostraca | | Chydoridae | *Alona costata* | | <5 |
| Branchiopoda | Diplostraca | | Chydoridae | *Alona guttata* | | <5 |
| Branchiopoda | Diplostraca | | Chydoridae | *Alona intermedia* | | lacking |
| Branchiopoda | Diplostraca | | Chydoridae | *Alona lepida* | | lacking |
| Branchiopoda | Diplostraca | | Chydoridae | *Alona quadrangularis* | | <5 |
| Branchiopoda | Diplostraca | | Chydoridae | *Alona rectangula* | | <5 |
| Branchiopoda | Diplostraca | | Chydoridae | *Alona rustica* | | lacking |
| Branchiopoda | Diplostraca | | Chydoridae | *Alona setulosa* | | 5to25 |
| Branchiopoda | Diplostraca | | Chydoridae | *Alonella excisa*  (in BOLD as Alona cf. excisia) | | 5to25 |
| Branchiopoda | Diplostraca | | Chydoridae | *Alonella exigua* | | <5 |
| Branchiopoda | Diplostraca | | Chydoridae | *Alonella nana* | | lacking |
| Branchiopoda | Diplostraca | | Chydoridae | *Alonopsis elongata* | | 5to25 |
| Branchiopoda | Diplostraca | | Chydoridae | *Anchistropus minor* | | lacking |
| Branchiopoda | Diplostraca | | Chydoridae | *Camptocercus macrurus* | | lacking |
| Branchiopoda | Diplostraca | | Chydoridae | *Camptocercus rectirostris* | | 5to25 |
| Branchiopoda | Diplostraca | | Chydoridae | *Chydorus faviformis* | | <5 |
| Branchiopoda | Diplostraca | | Chydoridae | *Chydorus gibbus* | | lacking |
| Branchiopoda | Diplostraca | | Chydoridae | *Chydorus latus* | | lacking |
| Branchiopoda | Diplostraca | | Chydoridae | *Chydorus sphaericus* | | 5to25 |
| Branchiopoda | Diplostraca | | Chydoridae | *Disparalona acutirostris* | | <5 |
| Branchiopoda | Diplostraca | | Chydoridae | *Disparalona hamata* | | lacking |
| Branchiopoda | Diplostraca | | Chydoridae | *Disparalona leei* | | lacking |
| Branchiopoda | Diplostraca | | Chydoridae | *Disparalona rostrata* | | <5 |
| Branchiopoda | Diplostraca | | Chydoridae | *Dunhevedia crassa* | | 5to25 |
| Branchiopoda | Diplostraca | | Chydoridae | *Eurycercus lamellatus* | | 5to25 |
| Branchiopoda | Diplostraca | | Chydoridae | *Graptoleberis testudinaria* | | 5to25 |
| Branchiopoda | Diplostraca | | Chydoridae | *Kurzia latissima* | | <5 |
| Branchiopoda | Diplostraca | | Chydoridae | *Leydigia acanthocercoides* | | <5 |
| Branchiopoda | Diplostraca | | Chydoridae | *Leydigia leydigi* | | lacking |
| Branchiopoda | Diplostraca | | Chydoridae | *Leydigia quadrangularis* | | zero |
| Branchiopoda | Diplostraca | | Chydoridae | *Monospilus dispar* | | 5to25 |
| Branchiopoda | Diplostraca | | Chydoridae | *Notoalona globulosa* | | lacking |
| Branchiopoda | Diplostraca | | Chydoridae | *Picripleuroxus denticulatus*  (in BOLD as Pleuroxus denticulatus) | | 5to25 |
| Branchiopoda | Diplostraca | | Chydoridae | *Picripleuroxus laevis* | | lacking |
| Branchiopoda | Diplostraca | | Chydoridae | *Picripleuroxus striatus* | | 5to25 |
| Branchiopoda | Diplostraca | | Chydoridae | *Pleuroxus aduncus* | | 5to25 |
| Branchiopoda | Diplostraca | | Chydoridae | *Pleuroxus procurvus* | | 5to25 |
| Branchiopoda | Diplostraca | | Chydoridae | *Pleuroxus trigonellus* | | lacking |
| Branchiopoda | Diplostraca | | Chydoridae | *Pleuroxus truncatus* | | 5to25 |
| Branchiopoda | Diplostraca | | Chydoridae | *Pleuroxus uncinatus* | | zero |
| Branchiopoda | Diplostraca | | Chydoridae | *Pseudochydorus globosus* | | 5to25 |
| Branchiopoda | Diplostraca | | Chydoridae | *Rhynchotalona falcata* | | <5 |
| Branchiopoda | Diplostraca | | Daphniidae | *Ceriodaphnia lacustris* | | 5to25 |
| Branchiopoda | Diplostraca | | Daphniidae | *Ceriodaphnia laticaudata* | | <5 |
| Branchiopoda | Diplostraca | | Daphniidae | *Ceriodaphnia megops*  (in BOLD as C. megalops) | | 5to25 |
| Branchiopoda | Diplostraca | | Daphniidae | *Ceriodaphnia pulchella* | | zero |
| Branchiopoda | Diplostraca | | Daphniidae | *Ceriodaphnia quadrangula* | | zero |
| Branchiopoda | Diplostraca | | Daphniidae | *Ceriodaphnia reticulata* | | <5 |
| Branchiopoda | Diplostraca | | Daphniidae | *Daphnia ambigua* | | >25 |
| Branchiopoda | Diplostraca | | Daphniidae | ***Daphnia galeata*** | | 5to25 |
| Branchiopoda | Diplostraca | | Daphniidae | *Daphnia laevis* | | >25 |
| Branchiopoda | Diplostraca | | Daphniidae | *Daphnia longiremis* | | 5to25 |
| Branchiopoda | Diplostraca | | Daphniidae | ***Daphnia lumholtzi*** | | 5to25 |
| Branchiopoda | Diplostraca | | Daphniidae | *Daphnia parvula* | | 5to25 |
| Branchiopoda | Diplostraca | | Daphniidae | *Daphnia pulex* | | >25 |
| Branchiopoda | Diplostraca | | Daphniidae | *Daphnia pulicaria* | | >25 |
| Branchiopoda | Diplostraca | | Daphniidae | *Daphnia retrocurva* | | <5 |
| Branchiopoda | Diplostraca | | Daphniidae | *Daphnia schodleri* | | <5 |
| Branchiopoda | Diplostraca | | Daphniidae | *Scapholeberis aurita* | | lacking |
| Branchiopoda | Diplostraca | | Daphniidae | *Scapholeberis kingi* | | lacking |
| Branchiopoda | Diplostraca | | Daphniidae | *Simocephalus exspinosus* | | 5to25 |
| Branchiopoda | Diplostraca | | Daphniidae | *Simocephalus serrulatus* | | 5to25 |
| Branchiopoda | Diplostraca | | Daphniidae | *Simocephalus vetulus* | | 5to25 |
| Branchiopoda | Diplostraca | | Holopediidae | *Holopedium gibberum* | | 5to25 |
| Branchiopoda | Diplostraca | | Ilyocryptidae | *Ilyocryptus acutifrons* | | zero |
| Branchiopoda | Diplostraca | | Ilyocryptidae | *Ilyocryptus sordidus* | | 5to25 |
| Branchiopoda | Diplostraca | | Ilyocryptidae | *Ilyocryptus spinifer* | | 5to25 |
| Branchiopoda | Diplostraca | | Leptodoridae | *Leptodora kindtii* | | >25 |
| Branchiopoda | Diplostraca | | Macrothricidae | *Acantholeberis curvirostris* | | <5 |
| Branchiopoda | Diplostraca | | Macrothricidae | *Bunops serricaudata* | | lacking |
| Branchiopoda | Diplostraca | | Macrothricidae | *Drepanothrix dentata* | | <5 |
| Branchiopoda | Diplostraca | | Macrothricidae | *Lathonura rectirostris* | | <5 |
| Branchiopoda | Diplostraca | | Macrothricidae | *Macrothrix laticornis* | | lacking |
| Branchiopoda | Diplostraca | | Macrothricidae | *Macrothrix rosea* | | lacking |
| Branchiopoda | Diplostraca | | Macrothricidae | *Ophryoxus gracilis* | | 5to25 |
| Branchiopoda | Diplostraca | | Macrothricidae | *Streblocerus serricaudatus*  (in BOLD as S. serricaudis) | | <5 |
| Branchiopoda | Diplostraca | | Macrothricidae | *Wlassicsia kinistinensis* | | lacking |
| Branchiopoda | Diplostraca | | Moinidae | *Moina macrocopa* | | >25 |
| Branchiopoda | Diplostraca | | Moinidae | *Moina micrura* | | 5to25 |
| Branchiopoda | Diplostraca | | Polyphemoidea | *Polyphemus pediculus* | | >25 |
| Branchiopoda | Diplostraca | | Sididae | *Diaphanosoma birgei* | | 5to25 |
| Branchiopoda | Diplostraca | | Sididae | *Diaphanosoma brachyurum* | | 5to25 |
| Branchiopoda | Diplostraca | | Sididae | *Diaphanosoma leuchtenbergianum* | | lacking |
| Branchiopoda | Diplostraca | | Sididae | *Latona parviremis* | | lacking |
| Branchiopoda | Diplostraca | | Sididae | *Latona setifera* | | zero |
| Branchiopoda | Diplostraca | | Sididae | *Latonopsis occidentalis* | | lacking |
| Branchiopoda | Diplostraca | | Sididae | *Sida crystallina* | | >25 |
| Malacostraca | Amphipoda | | Crangonyctidae | Crangonyx gracilis | | <5 |
| Malacostraca | Amphipoda | | Crangonyctidae | Crangonyx pseudogracilis | | 5to25 |
| Malacostraca | Amphipoda | | Gammaridae | **Echinogammarus ischnus** | | 5to25 |
| Malacostraca | Amphipoda | | Gammaridae | **Gammarus fasciatus** | | >25 |
| Malacostraca | Amphipoda | | Gammaridae | Gammarus limnaeus | | lacking |
| Malacostraca | Amphipoda | | Gammaridae | Gammarus pseudolimnaeus | | <5 |
| Malacostraca | Amphipoda | | Gammaridae | **Gammarus tigrinus** | | >25 |
| Malacostraca | Amphipoda | | Hyalellidae | Hyalella azteca | | >25 |
| Malacostraca | Amphipoda | | Pontoporeiidae | *Diporeia brevicornis* | | lacking |
| Malacostraca | Amphipoda | | Pontoporeiidae | *Diporeia erythrophthalma* | | lacking |
| Malacostraca | Amphipoda | | Pontoporeiidae | *Diporeia filicornis* | | lacking |
| Malacostraca | Amphipoda | | Pontoporeiidae | *Diporeia hoyi* | | >25 |
| Malacostraca | Amphipoda | | Pontoporeiidae | *Diporeia intermedia* | | lacking |
| Malacostraca | Amphipoda | | Pontoporeiidae | *Diporeia kendalli* | | lacking |
| Malacostraca | Amphipoda | | Pontoporeiidae | *Pontoporeia affinis* | | lacking |
| Malacostraca | Decapoda | | Cambaridae | Cambarus bartonii | | <5 |
| Malacostraca | Decapoda | | Cambaridae | Cambarus diogenes | | lacking |
| Malacostraca | Decapoda | | Cambaridae | Cambarus robustus | | lacking |
| Malacostraca | Decapoda | | Cambaridae | Cambarus thomai | | lacking |
| Malacostraca | Decapoda | | Cambaridae | Fallicambarus fodiens | | 5to25 |
| Malacostraca | Decapoda | | Cambaridae | Orconectes immunis | | <5 |
| Malacostraca | Decapoda | | Cambaridae | **Orconectes obscurus** | | <5 |
| Malacostraca | Decapoda | | Cambaridae | Orconectes propinquus | | 5to25 |
| Malacostraca | Decapoda | | Cambaridae | **Orconectes rusticus** | | 5to25 |
| Malacostraca | Decapoda | | Cambaridae | Orconectes sanbornii | | <5 |
| Malacostraca | Decapoda | | Cambaridae | Orconectes virilis | | 5to25 |
| Malacostraca | Decapoda | | Cambaridae | Procambarus acutus | | <5 |
| Malacostraca | Decapoda | | Cambaridae | **Procambarus clarkii** | | >25 |
| Malacostraca | Decapoda | | Palaemonidae | Palaemonetes kadiakensis | | lacking |
| Malacostraca | Isopoda | | Asellidae | Caecidotea intermedia | | lacking |
| Malacostraca | Isopoda | | Asellidae | Caecidotea racovitzai | | <5 |
| Malacostraca | Isopoda | | Asellidae | Lirceus lineatus | | <5 |
| Malacostraca | Mysida | | Mysidae | **Hemimysis anomala** | | 5to25 |
| Malacostraca | Mysida | | Mysidae | *Mysis diluviana* | | >25 |
| Maxillopoda | Arguloida | | Argulidae | ***Argulus japonicus*** | | lacking |
| Maxillopoda | Calanoida | | Aetideidae | *Senecella calanoides* | | <5 |
| Maxillopoda | Calanoida | | Diaptomidae | *Diaptomus reighardi* (in BOLD as Skistodiaptomus reighardi) | | <5 |
| Maxillopoda | Calanoida | | Diaptomidae | *Diaptomus sicilis* | | lacking |
| Maxillopoda | Calanoida | | Temoridae | *Epischura lacustris* | | >25 |
| Maxillopoda | Calanoida | | Temoridae | ***Eurytemora affinis*** | | >25 |
| Maxillopoda | Cyclopoida | | Cyclopidae | *Acanthocyclops vernalis* | | 5to25 |
| Maxillopoda | Cyclopoida | | Cyclopidae | *Cyclops bicuspidatus* | | <5 |
| Maxillopoda | Cyclopoida | | Cyclopidae | *Cyclops scutifer* | | lacking |
| Maxillopoda | Cyclopoida | | Cyclopidae | ***Cyclops strenuus*** | | <5 |
| Maxillopoda | Cyclopoida | | Cyclopidae | *Cyclops varicans* | | lacking |
| Maxillopoda | Cyclopoida | | Cyclopidae | *Cyclops vernalis* | | lacking |
| Maxillopoda | Cyclopoida | | Cyclopidae | *Diacyclops nanus* | | zero |
| Maxillopoda | Cyclopoida | | Cyclopidae | *Diacyclops navus* | | lacking |
| Maxillopoda | Cyclopoida | | Cyclopidae | *Diacyclops nearcticus* | | lacking |
| Maxillopoda | Cyclopoida | | Cyclopidae | *Diacyclops thomasi* | | <5 |
| Maxillopoda | Cyclopoida | | Cyclopidae | *Ectocyclops phaleratus* | | lacking |
| Maxillopoda | Cyclopoida | | Cyclopidae | *Eucyclops agilis* | | zero |
| Maxillopoda | Cyclopoida | | Cyclopidae | *Eucyclops elegans* | | lacking |
| Maxillopoda | Cyclopoida | | Cyclopidae | *Eucyclops prionophorus* | | zero |
| Maxillopoda | Cyclopoida | | Cyclopidae | *Eucyclops speratus* | | 5to25 |
| Maxillopoda | Cyclopoida | | Cyclopidae | *Macrocyclops albidus* | | 5to25 |
| Maxillopoda | Cyclopoida | | Cyclopidae | ***Megacyclops viridis*** | | <5 |
| Maxillopoda | Cyclopoida | | Cyclopidae | *Mesocyclops edax* | | >25 |
| Maxillopoda | Cyclopoida | | Cyclopidae | *Microcyclops rubellus* | | <5 |
| Maxillopoda | Cyclopoida | | Cyclopidae | *Microcyclops varicans* | | lacking |
| Maxillopoda | Cyclopoida | | Cyclopidae | Orthocyclops modestus | | lacking |
| Maxillopoda | Cyclopoida | | Cyclopidae | *Paracyclops chiltoni* (was subspecies of P. fimbriatus which is in BOLD) | | lacking |
| Maxillopoda | Cyclopoida | | Cyclopidae | *Paracyclops fimbriatus* | | 5to25 |
| Maxillopoda | Cyclopoida | | Cyclopidae | *Tropocyclops prasinus* | | 5to25 |
| Maxillopoda | Diplostraca | | Aetideidae | *Senecella calanoides* | | <5 |
| Maxillopoda | Diplostraca | | Centropagidae | *Limnocalanus macrurus* | | >25 |
| Maxillopoda | Diplostraca | | Centropagidae | *Osphranticum labronectum* | | <5 |
| Maxillopoda | Diplostraca | | Diaptomidae | *Leptodiaptomus ashlandi* | | <5 |
| Maxillopoda | Diplostraca | | Diaptomidae | *Leptodiaptomus minutus* | | >25 |
| Maxillopoda | Diplostraca | | Diaptomidae | *Leptodiaptomus sicilis* | | 5to25 |
| Maxillopoda | Diplostraca | | Diaptomidae | *Leptodiaptomus siciloides* | | 5to25 |
| Maxillopoda | Diplostraca | | Diaptomidae | *Onychodiaptomus birgei* | | lacking |
| Maxillopoda | Diplostraca | | Diaptomidae | *Skistodiaptomus oregonensis* | | 5to25 |
| Maxillopoda | Diplostraca | | Diaptomidae | ***Skistodiaptomus pallidus*** | | >25 |
| Maxillopoda | Diplostraca | | Diaptomidae | *Skistodiaptomus reighardi* | | <5 |
| Maxillopoda | Harpacticoida | | Ameiridae | ***Nitokra hibernica*** | | lacking |
| Maxillopoda | Harpacticoida | | Ameiridae | ***Nitokra incerta*** | | lacking |
| Maxillopoda | Harpacticoida | | Ameiridae | *Nitokra spinipes* | | lacking |
| Maxillopoda | Harpacticoida | | Canthocamptidae | *Attheyella illinoisensis* | | lacking |
| Maxillopoda | Harpacticoida | | Canthocamptidae | *Attheyella nordenskioldi* | | lacking |
| Maxillopoda | Harpacticoida | | Canthocamptidae | *Bryocamptus nivalis* | | lacking |
| Maxillopoda | Harpacticoida | | Canthocamptidae | *Bryocamptus zschokkei* | | lacking |
| Maxillopoda | Harpacticoida | | Canthocamptidae | *Canthocamptus robertcokeri* | | genus |
| Maxillopoda | Harpacticoida | | Canthocamptidae | ***Canthocamptus staphylinoides*** | | genus |
| Maxillopoda | Harpacticoida | | Canthocamptidae | *Epactophanes richardi* | | lacking |
| Maxillopoda | Harpacticoida | | Canthocamptidae | ***Heteropsyllus nunni*** | | lacking |
| Maxillopoda | Harpacticoida | | Canthocamptidae | *Mesochra alaskana* | | lacking |
| Maxillopoda | Harpacticoida | | Canthocamptidae | *Moraria cristata* | | lacking |
| Maxillopoda | Harpacticoida | | Diosaccidae | ***Schizopera borutzkyi*** | | lacking |
| Maxillopoda | Harpacticoida | | Laophontidae | ***Onychocamptus mohammed*** | | lacking |
| Maxillopoda | Harpacticoida | | Parastenocarididae | *Parastenocaris delamarei* | | lacking |
| Maxillopoda | Harpacticoida | | Parastenocarididae | *Parastenocaris lacustris* | | lacking |
| Maxillopoda | Poecilostomatoida | | Ergasilidae | ***Neoergasilus japonicus*** | | lacking |
| Ostracoda | Podocopida | | Candonidae | *Candona simpsoni* | | lacking |
| Ostracoda | Podocopida | | Cyprididae | *Cypria maculata* | | lacking |
| Ostracoda | Podocopida | | Cyprididae | *Cypria ophtalmica* | | 5to25 |
| Ostracoda | Podocopida | | Cyprididae | *Cypria pellucida* | | lacking |
| Ostracoda | Podocopida | | Cyprididae | *Cypridopsis vidua* | | >25 |
| Ostracoda | Podocopida | | Cyprididae | *Physocypria pustulosa* | | lacking |
| Ostracoda | Podocopida | | Darwinulidae | *Darwinula stevensoni* | | <5 |
| **Phylum *Arthropoda - Hexapoda*** |  | |  |  | |  |
| Entognatha | Collembola | | Poduridae | Podura aquatica | | 5to25 |
| Insecta | Coleoptera | | Chrysomelidae | Neohaemonia flagellata | | zero |
| Insecta | Coleoptera | | Chrysomelidae | Neohaemonia melsheimeri | | <5 |
| Insecta | Coleoptera | | Chrysomelidae | Neohaemonia minnesotensis | | zero |
| Insecta | Coleoptera | | Chrysomelidae | Neohaemonia nigricornis | | zero |
| Insecta | Coleoptera | | Chrysomelidae | Prasocuris phellandrii | | 5to25 |
| Insecta | Coleoptera | | Dytiscidae | Acilius sylvanus | | <5 |
| Insecta | Coleoptera | | Dytiscidae | Dytiscus fasciventris | | 5to25 |
| Insecta | Coleoptera | | Dytiscidae | Heterosternuta wickhami | | <5 |
| Insecta | Coleoptera | | Dytiscidae | Hydroporus niger | | zero |
| Insecta | Coleoptera | | Dytiscidae | Hygrotus dissimilis | | zero |
| Insecta | Coleoptera | | Dytiscidae | Hygrotus impressopunctatus | | 5to25 |
| Insecta | Coleoptera | | Dytiscidae | Hygrotus sayi | | <5 |
| Insecta | Coleoptera | | Dytiscidae | Laccophilus maculosus | | zero |
| Insecta | Coleoptera | | Elmidae | Ancyronyx variegatus  (in BOLD as Ancyronyx variegata) | | <5 |
| Insecta | Coleoptera | | Elmidae | Dubiraphia bivittata | | <5 |
| Insecta | Coleoptera | | Elmidae | Macronychus glabratus | | 5to25 |
| Insecta | Coleoptera | | Elmidae | Optioservus fastiditus | | 5to25 |
| Insecta | Coleoptera | | Elmidae | Stenelmis crenata | | >25 |
| Insecta | Coleoptera | | Elmidae | Stenelmis quadrimaculata | | lacking |
| Insecta | Coleoptera | | Erirhinidae | **Tanysphyrus lemnae** | | 5to25 |
| Insecta | Coleoptera | | Gyrinidae | Gyrinus confinis | | zero |
| Insecta | Coleoptera | | Haliplidae | Haliplus borealis | | zero |
| Insecta | Coleoptera | | Haliplidae | Haliplus immaculicollis | | 5to25 |
| Insecta | Coleoptera | | Haliplidae | Haliplus triopsis | | zero |
| Insecta | Coleoptera | | Haliplidae | Peltodytes duodecimpuntatus | | zero |
| Insecta | Coleoptera | | Haliplidae | Peltodytes edentulus | | zero |
| Insecta | Coleoptera | | Haliplidae | Peltodytes lengi | | zero |
| Insecta | Coleoptera | | Haliplidae | Peltodytes muticus | | zero |
| Insecta | Coleoptera | | Haliplidae | Peltodytes sexmaculatus | | lacking |
| Insecta | Coleoptera | | Hydrophilidae | Anacaena limbata | | <5 |
| Insecta | Coleoptera | | Hydrophilidae | Anacaena lutescens | | 5to25 |
| Insecta | Coleoptera | | Hydrophilidae | Berosus fraternus | | <5 |
| Insecta | Coleoptera | | Hydrophilidae | Berosus infuscatus | | lacking |
| Insecta | Coleoptera | | Hydrophilidae | Berosus peregrinus | | 5to25 |
| Insecta | Coleoptera | | Hydrophilidae | Berosus sayi | | 5to25 |
| Insecta | Coleoptera | | Hydrophilidae | Enochrus fimbriatus | | zero |
| Insecta | Coleoptera | | Hydrophilidae | Enochrus hamiltoni | | 5to25 |
| Insecta | Coleoptera | | Hydrophilidae | Enochrus ochraceus | | <5 |
| Insecta | Coleoptera | | Hydrophilidae | Enochrus pygmaeus | | zero |
| Insecta | Coleoptera | | Hydrophilidae | Enochrus sayi | | zero |
| Insecta | Coleoptera | | Hydrophilidae | Helophorus lineatus | | zero |
| Insecta | Coleoptera | | Hydrophilidae | Helophorus marginicollis | | zero |
| Insecta | Coleoptera | | Hydrophilidae | Helophorus orientalis | | <5 |
| Insecta | Coleoptera | | Hydrophilidae | Hydrobius fuscipes | | >25 |
| Insecta | Coleoptera | | Hydrophilidae | Hydrochara leechi | | lacking |
| Insecta | Coleoptera | | Hydrophilidae | Paracymus subcupreus | | zero |
| Insecta | Coleoptera | | Hydrophilidae | Tropisternus lateralis | | 5to25 |
| Insecta | Coleoptera | | Hydrophilidae | Tropisternus natator | | <5 |
| Insecta | Coleoptera | | Lutrochidae | Lutrochus laticeps | | zero |
| Insecta | Coleoptera | | Noteridae | Hydrocanthus iricolor | | 5to25 |
| Insecta | Coleoptera | | Psephenidae | Ectopria leechi | | zero |
| Insecta | Coleoptera | | Psephenidae | Ectopria nervosa | | zero |
| Insecta | Coleoptera | | Psephenidae | Psephenus herricki | | >25 |
| Insecta | Diptera | | Ceratopogonidae | Sphaeromias longipennis | | lacking |
| Insecta | Diptera | | Chaoboridae | Chaoborus flavicans | | <5 |
| Insecta | Diptera | | Chaoboridae | Chaoborus punctipennis | | zero |
| Insecta | Diptera | | Chironomidae | Ablabesmyia annulata | | lacking |
| Insecta | Diptera | | Chironomidae | Ablabesmyia monilis | | 5to25 |
| Insecta | Diptera | | Chironomidae | Ablabesmyia mallochi | | lacking |
| Insecta | Diptera | | Chironomidae | Chironomus decorus | | >25 |
| Insecta | Diptera | | Chironomidae | Chironomus halophilus | | lacking |
| Insecta | Diptera | | Chironomidae | Chironomus plumosus | | 5to25 |
| Insecta | Diptera | | Chironomidae | Chironomus riparius | | >25 |
| Insecta | Diptera | | Chironomidae | Chironomus staegeri | | <5 |
| Insecta | Diptera | | Chironomidae | Chironomus tentans | | 5to25 |
| Insecta | Diptera | | Chironomidae | Chironomus anthracinus | | lacking |
| Insecta | Diptera | | Chironomidae | Cladotanytarsus mancus | | 5to25 |
| Insecta | Diptera | | Chironomidae | Cladotanytarsus vanderwulpi  (in BOLD but not ITIS) | | <5 |
| Insecta | Diptera | | Chironomidae | Coelotanypus concinnus | | lacking |
| Insecta | Diptera | | Chironomidae | Corynoneura lacustris | | <5 |
| Insecta | Diptera | | Chironomidae | Corynoneura lobata | | 5to25 |
| Insecta | Diptera | | Chironomidae | Cricotopus bicinctus | | >25 |
| Insecta | Diptera | | Chironomidae | Cricotopus festivellus | | 5to25 |
| Insecta | Diptera | | Chironomidae | Cricotopus ornatus | | 5to25 |
| Insecta | Diptera | | Chironomidae | Cricotopus sylvestris | | >25 |
| Insecta | Diptera | | Chironomidae | Cricotopus trifascia | | 5to25 |
| Insecta | Diptera | | Chironomidae | Cricotopus vierriensis | | 5to25 |
| Insecta | Diptera | | Chironomidae | Cricotopus cylindraceus | | lacking |
| Insecta | Diptera | | Chironomidae | Cricotopus festivellus | | 5to25 |
| Insecta | Diptera | | Chironomidae | Cricotopus fuscus | | lacking |
| Insecta | Diptera | | Chironomidae | Cricotopus sylvestris | | >25 |
| Insecta | Diptera | | Chironomidae | Cricotopus tremulus | | >25 |
| Insecta | Diptera | | Chironomidae | Cricotopus trifascia | | 5to25 |
| Insecta | Diptera | | Chironomidae | Cryptochironomus digitatus | | lacking |
| Insecta | Diptera | | Chironomidae | Cryptochironomus fulvus | | <5 |
| Insecta | Diptera | | Chironomidae | Dicrotendipes fumidus | | lacking |
| Insecta | Diptera | | Chironomidae | Dicrotendipes modestus | | >25 |
| Insecta | Diptera | | Chironomidae | Dicrotendipes neomodestus | | lacking |
| Insecta | Diptera | | Chironomidae | Dicrotendipes nervosus | | 5to25 |
| Insecta | Diptera | | Chironomidae | Diplocladius cultriger | | 5to25 |
| Insecta | Diptera | | Chironomidae | Endochironomus subtendens  (in BOLD as Tanytarsus subtendens) | | lacking |
| Insecta | Diptera | | Chironomidae | Epiococladius flavens | | lacking |
| Insecta | Diptera | | Chironomidae | Glyptotendipes loberiferus | | 5to25 |
| Insecta | Diptera | | Chironomidae | Guttipelopia guttipennis | | <5 |
| Insecta | Diptera | | Chironomidae | Guttipelopia rosenbergi | | lacking |
| Insecta | Diptera | | Chironomidae | Halocladius variabilis | | >25 |
| Insecta | Diptera | | Chironomidae | Halocladius vitripennis | | lacking |
| Insecta | Diptera | | Chironomidae | Harnischia curtilamellata | | <5 |
| Insecta | Diptera | | Chironomidae | Heterotrissocladius changi | | >25 |
| Insecta | Diptera | | Chironomidae | Heterotrissocladius marcidus | | 5to25 |
| Insecta | Diptera | | Chironomidae | Heterotrissocladius oliveri | | lacking |
| Insecta | Diptera | | Chironomidae | Heterotrissocladius subpilosa gr. | | <5 |
| Insecta | Diptera | | Chironomidae | Hydrobaenus pilipes | | lacking |
| Insecta | Diptera | | Chironomidae | Kiefferulus dux | | <5 |
| Insecta | Diptera | | Chironomidae | Krenosmittia boreoalpina | | <5 |
| Insecta | Diptera | | Chironomidae | Krenosmittia camptophleps | | 5to25 |
| Insecta | Diptera | | Chironomidae | Labrundinia pilosilla | | lacking |
| Insecta | Diptera | | Chironomidae | Microchironomus nigrovittatus | | lacking |
| Insecta | Diptera | | Chironomidae | Micropsectra politans | | >25 |
| Insecta | Diptera | | Chironomidae | Microtendipes caelum | | lacking |
| Insecta | Diptera | | Chironomidae | Microtendipes pedellus | | >25 |
| Insecta | Diptera | | Chironomidae | Monodiamesa depectinata | | lacking |
| Insecta | Diptera | | Chironomidae | Monodiamesa tuberculata | | lacking |
| Insecta | Diptera | | Chironomidae | Monopelopia boliekae | | lacking |
| Insecta | Diptera | | Chironomidae | Monopelopia tillandsia | | lacking |
| Insecta | Diptera | | Chironomidae | Nilotanypus fimbriatus | | 5to25 |
| Insecta | Diptera | | Chironomidae | Nilotanypus kansensis | | lacking |
| Insecta | Diptera | | Chironomidae | Orthocladius obumbratus | | lacking |
| Insecta | Diptera | | Chironomidae | Pagastia orthogonia | | 5to25 |
| Insecta | Diptera | | Chironomidae | Pagastia partica | | lacking |
| Insecta | Diptera | | Chironomidae | Parachironomus abortivus | | lacking |
| Insecta | Diptera | | Chironomidae | Parachironomus arcuatus | | <5 |
| Insecta | Diptera | | Chironomidae | Parachironomus chaetaolus | | lacking |
| Insecta | Diptera | | Chironomidae | Paracladius conversus | | zero |
| Insecta | Diptera | | Chironomidae | Paracladopelma camptolabis | | <5 |
| Insecta | Diptera | | Chironomidae | Paracladopelma doris | | lacking |
| Insecta | Diptera | | Chironomidae | Paracladopelma nais | | lacking |
| Insecta | Diptera | | Chironomidae | Paracladopelma nigritula gr. | | lacking |
| Insecta | Diptera | | Chironomidae | Paracladopelma undine | | <5 |
| Insecta | Diptera | | Chironomidae | Paracladopelma winnelli | | >25 |
| Insecta | Diptera | | Chironomidae | Parakiefferiella nigra | | lacking |
| Insecta | Diptera | | Chironomidae | Paralauterborniella nigrohalterale  (in BOLD as P. nigrohalteralis) | | 5to25 |
| Insecta | Diptera | | Chironomidae | Parochlus kiefferi | | 5to25 |
| Insecta | Diptera | | Chironomidae | Polypedilum fallax | | lacking |
| Insecta | Diptera | | Chironomidae | Polypedilum flavum  (species not in ITIS) | | 5to25 |
| Insecta | Diptera | | Chironomidae | Polypedilum halterale | | lacking |
| Insecta | Diptera | | Chironomidae | Polypedilum illinoense | | lacking |
| Insecta | Diptera | | Chironomidae | Polypedilum laetum | | lacking |
| Insecta | Diptera | | Chironomidae | Polypedilum scalaenum | | 5to25 |
| Insecta | Diptera | | Chironomidae | Polypedilum simulans | | 5to25 |
| Insecta | Diptera | | Chironomidae | Potthastia longimana  (in BOLD as P. longimanus) | | <5 |
| Insecta | Diptera | | Chironomidae | Procladius sublettei | | lacking |
| Insecta | Diptera | | Chironomidae | Prodiamesa cubita | | lacking |
| Insecta | Diptera | | Chironomidae | Prodiamesa olivacea | | 5to25 |
| Insecta | Diptera | | Chironomidae | Protanypus ramosus | | lacking |
| Insecta | Diptera | | Chironomidae | Psectrocladius psilopterus | | <5 |
| Insecta | Diptera | | Chironomidae | Psectrocladius simulans | | <5 |
| Insecta | Diptera | | Chironomidae | Psectrocladius sordidellus | | <5 |
| Insecta | Diptera | | Chironomidae | Pseudochironomus articaudus | | lacking |
| Insecta | Diptera | | Chironomidae | Pseudochironomus fulviventris | | lacking |
| Insecta | Diptera | | Chironomidae | Rheotanytarsus exiguus | | lacking |
| Insecta | Diptera | | Chironomidae | Robackia demeijerei | | lacking |
| Insecta | Diptera | | Chironomidae | Saetheria tylus | | <5 |
| Insecta | Diptera | | Chironomidae | Stempellina bausei | | 5to25 |
| Insecta | Diptera | | Chironomidae | Stempellinella brevis | | 5to25 |
| Insecta | Diptera | | Chironomidae | Stempellinella minor | | lacking |
| Insecta | Diptera | | Chironomidae | Stempellinella saltuum | | <5 |
| Insecta | Diptera | | Chironomidae | Synorthocladius semivirens | | 5to25 |
| Insecta | Diptera | | Chironomidae | Tanypus neopunctipennis | | lacking |
| Insecta | Diptera | | Chironomidae | Tanytarsus glabrescens | | >25 |
| Insecta | Diptera | | Chironomidae | Thienenmannimyia senata | | lacking |
| Insecta | Diptera | | Chironomidae | Tribelos jucundus | | lacking |
| Insecta | Diptera | | Chironomidae | Xenochironomus festivus | | lacking |
| Insecta | Diptera | | Chironomidae | Xenochironomus taenionotus | | lacking |
| Insecta | Diptera | | Chironomidae | Xenochironomus xenolabis | | 5to25 |
| Insecta | Diptera | | Chironomidae | Zavrelia pentatoma | | <5 |
| Insecta | Diptera | | Culicidae | Aedes infirmatus | | 5to25 |
| Insecta | Diptera | | Culicidae | Aedes vexans | | >25 |
| Insecta | Diptera | | Culicidae | Uranotaenia sapphirina | | 5to25 |
| Insecta | Diptera | | Dixidae | Dixa modesta | | lacking |
| Insecta | Ephemeroptera | | Baetiscidae | Baetisca bajkovi | | lacking |
| Insecta | Ephemeroptera | | Baetiscidae | Baetisca obesa | | <5 |
| Insecta | Ephemeroptera | | Caenidae | Caenis amica | | 5to25 |
| Insecta | Ephemeroptera | | Caenidae | Caenis latipennis | | >25 |
| Insecta | Ephemeroptera | | Caenidae | Caenis youngi | | >25 |
| Insecta | Ephemeroptera | | Ephemerellidae | Dannella simplex | | 5to25 |
| Insecta | Ephemeroptera | | Ephemerellidae | Dentatella coxalis | | <5 |
| Insecta | Ephemeroptera | | Ephemerellidae | Ephemerella invaria | | >25 |
| Insecta | Ephemeroptera | | Ephemerellidae | Ephemerella subvaria | | >25 |
| Insecta | Ephemeroptera | | Ephemerellidae | Eurylophella aestiva | | >25 |
| Insecta | Ephemeroptera | | Ephemerellidae | Eurylophella bicolor | | >25 |
| Insecta | Ephemeroptera | | Ephemerellidae | Eurylophella lululenta | | <5 |
| Insecta | Ephemeroptera | | Ephemerellidae | Eurylophella temporalis | | 5to25 |
| Insecta | Ephemeroptera | | Ephemerellidae | Teloganopsis deficiens | | >25 |
| Insecta | Ephemeroptera | | Ephemeridae | Ephemera simulans | | >25 |
| Insecta | Ephemeroptera | | Ephemeridae | Hexagenia limbata | | >25 |
| Insecta | Ephemeroptera | | Heptageniidae | Heptagenia flavescens | | 5to25 |
| Insecta | Ephemeroptera | | Heptageniidae | Heptagenia pulla | | >25 |
| Insecta | Ephemeroptera | | Heptageniidae | Leucrocuta aphrodite | | 5to25 |
| Insecta | Ephemeroptera | | Heptageniidae | Leucrocuta hebe | | >25 |
| Insecta | Ephemeroptera | | Heptageniidae | Leucrocuta juno | | <5 |
| Insecta | Ephemeroptera | | Heptageniidae | Leucrocuta maculipennis | | 5to25 |
| Insecta | Ephemeroptera | | Heptageniidae | Maccaffertium pulchellum | | 5to25 |
| Insecta | Ephemeroptera | | Heptageniidae | Maccaffertium terminatum | | >25 |
| Insecta | Ephemeroptera | | Heptageniidae | Maccaffertium vicarium | | >25 |
| Insecta | Ephemeroptera | | Heptageniidae | Nixe lucidipennis | | 5to25 |
| Insecta | Ephemeroptera | | Heptageniidae | Nixe perfida | | zero |
| Insecta | Ephemeroptera | | Heptageniidae | Stenacron interpunctatum | | >25 |
| Insecta | Ephemeroptera | | Heptageniidae | Stenonema femoratum | | >25 |
| Insecta | Ephemeroptera | | Isonychiidae | Isonychia sicca | | <5 |
| Insecta | Ephemeroptera | | Leptophlebiidae | Paraleptophlebia mollis | | >25 |
| Insecta | Ephemeroptera | | Leptophlebiidae | Paraleptophlebia ontario | | <5 |
| Insecta | Ephemeroptera | | Leptophlebiidae | Paraleptophlebia praepedita | | 5to25 |
| Insecta | Ephemeroptera | | Polymitarcyidae | Ephoron album | | 5to25 |
| Insecta | Hemiptera | | Belostomatidae | Belostoma flumineum | | 5to25 |
| Insecta | Hemiptera | | Corixidae | Callicorixa audeni | | >25 |
| Insecta | Hemiptera | | Corixidae | Corisella inscripta | | <5 |
| Insecta | Hemiptera | | Corixidae | Hesperocorixa atopodonta | | 5to25 |
| Insecta | Hemiptera | | Corixidae | Hesperocorixa lucida | | <5 |
| Insecta | Hemiptera | | Corixidae | Hesperocorixa michiganensis | | zero |
| Insecta | Hemiptera | | Corixidae | Hesperocorixa kennicotti | | <5 |
| Insecta | Hemiptera | | Corixidae | Palmacorixa buenoi | | <5 |
| Insecta | Hemiptera | | Corixidae | Palmacorixa nana | | lacking |
| Insecta | Hemiptera | | Corixidae | Sigara alternata | | 5to25 |
| Insecta | Hemiptera | | Corixidae | Sigara lineata | | zero |
| Insecta | Hemiptera | | Corixidae | Sigara modesta | | zero |
| Insecta | Hemiptera | | Corixidae | Sigara transfigurata | | zero |
| Insecta | Hemiptera | | Corixidae | Sigara trilineata | | <5 |
| Insecta | Hemiptera | | Corixidae | Sigara variabilis | | zero |
| Insecta | Hemiptera | | Corixidae | Trichocorixa borealis | | <5 |
| Insecta | Hemiptera | | Corixidae | Trichocorixa calva | | zero |
| Insecta | Hemiptera | | Corixidae | Trichocorixa kanza | | <5 |
| Insecta | Hemiptera | | Corixidae | Trichocorixa sexcincta | | 5to25 |
| Insecta | Hemiptera | | Gerridae | Aquarius remigis | | 5to25 |
| Insecta | Hemiptera | | Gerridae | Gerris buenoi | | 5to25 |
| Insecta | Hemiptera | | Gerridae | Gerris cornatus | | zero |
| Insecta | Hemiptera | | Gerridae | Gerris margniatus | | <5 |
| Insecta | Hemiptera | | Gerridae | Limnoporus canaliculatus | | zero |
| Insecta | Hemiptera | | Gerridae | Metrobates hesperius | | <5 |
| Insecta | Hemiptera | | Gerridae | Neogerris boninensis | | lacking |
| Insecta | Hemiptera | | Gerridae | Neogerris hesione | | lacking |
| Insecta | Hemiptera | | Hydrometridae | Hydrometra martini | | <5 |
| Insecta | Hemiptera | | Mesoveliidae | Mesovelia mulsanti | |  |
| Insecta | Hemiptera | | Nepidae | Ranatra fusca | | <5 |
| Insecta | Hemiptera | | Nepidae | Ranatra nigra | | <5 |
| Insecta | Hemiptera | | Notonectidae | Notonecta irrorata | | <5 |
| Insecta | Hemiptera | | Notonectidae | Notonecta raleighi | | lacking |
| Insecta | Hemiptera | | Pleidae | Neoplea striola | | <5 |
| Insecta | Lepidoptera | | Crambidae | Acentria nivea | | lacking |
| Insecta | Lepidoptera | | Crambidae | **Acentropus niveus** | | lacking |
| Insecta | Neuroptera | | Sisyridae | Climacia areolaris | | <5 |
| Insecta | Odonata | | Aeshnidae | Aeshna canadensis | | 5to25 |
| Insecta | Odonata | | Aeshnidae | Aeshna clepsydra | | lacking |
| Insecta | Odonata | | Aeshnidae | Aeshna constricta | | 5to25 |
| Insecta | Odonata | | Aeshnidae | Aeshna eremita | | >25 |
| Insecta | Odonata | | Aeshnidae | Aeshna interrupta | | >25 |
| Insecta | Odonata | | Aeshnidae | Aeshna juncea | | >25 |
| Insecta | Odonata | | Aeshnidae | Aeshna sitchensis | | 5to25 |
| Insecta | Odonata | | Aeshnidae | Aeshna subarctica | | 5to25 |
| Insecta | Odonata | | Aeshnidae | Aeshna tuberculifera | | 5to25 |
| Insecta | Odonata | | Aeshnidae | Aeshna umbrosa | | >25 |
| Insecta | Odonata | | Aeshnidae | Aeshna verticalis | | 5to25 |
| Insecta | Odonata | | Aeshnidae | Anax junius | | 5to25 |
| Insecta | Odonata | | Aeshnidae | Anax longipes | | <5 |
| Insecta | Odonata | | Aeshnidae | Basiaeschna janata | | 5to25 |
| Insecta | Odonata | | Aeshnidae | Boyeria grafiana | | >25 |
| Insecta | Odonata | | Aeshnidae | Boyeria vinosa | | 5to25 |
| Insecta | Odonata | | Aeshnidae | Epiaeschna heros | | <5 |
| Insecta | Odonata | | Aeshnidae | Gomphaeschna furcillata | | <5 |
| Insecta | Odonata | | Aeshnidae | Nasiaeschna pentacantha | | <5 |
| Insecta | Odonata | | Calopterygidae | Calopteryx aequabilis | | <5 |
| Insecta | Odonata | | Calopterygidae | Calopteryx angustipennis | | lacking |
| Insecta | Odonata | | Calopterygidae | Calopteryx maculata | | >25 |
| Insecta | Odonata | | Calopterygidae | Calopteryx amata | | zero |
| Insecta | Odonata | | Calopterygidae | Hetaerina americana | | <5 |
| Insecta | Odonata | | Calopterygidae | Hetaerina titia | | zero |
| Insecta | Odonata | | Coenagrionidae | Amphiagrion saucium | | <5 |
| Insecta | Odonata | | Coenagrionidae | Argia apicalis | | zero |
| Insecta | Odonata | | Coenagrionidae | Argia bipunctulata | | <5 |
| Insecta | Odonata | | Coenagrionidae | Argia fumipennis | | 5to25 |
| Insecta | Odonata | | Coenagrionidae | Argia moesta | | >25 |
| Insecta | Odonata | | Coenagrionidae | Argia sedula | | <5 |
| Insecta | Odonata | | Coenagrionidae | Argia tibialis | | <5 |
| Insecta | Odonata | | Coenagrionidae | Argia translata | | <5 |
| Insecta | Odonata | | Coenagrionidae | Chromagrion conditum | | 5to25 |
| Insecta | Odonata | | Coenagrionidae | Coenagrion interrogatum | | 5to25 |
| Insecta | Odonata | | Coenagrionidae | Coenagrion resolutum | | 5to25 |
| Insecta | Odonata | | Coenagrionidae | Enallagma anna | | zero |
| Insecta | Odonata | | Coenagrionidae | Enallagma antennatum | | 5to25 |
| Insecta | Odonata | | Coenagrionidae | Enallagma aspersum | | <5 |
| Insecta | Odonata | | Coenagrionidae | Enallagma basidens | | <5 |
| Insecta | Odonata | | Coenagrionidae | Enallagma boreale | | 5to25 |
| Insecta | Odonata | | Coenagrionidae | Enallagma carunculatum | | 5to25 |
| Insecta | Odonata | | Coenagrionidae | Enallagma civile | | >25 |
| Insecta | Odonata | | Coenagrionidae | Enallagma cyathigerum | | >25 |
| Insecta | Odonata | | Coenagrionidae | Enallagma divagans | | 5to25 |
| Insecta | Odonata | | Coenagrionidae | Enallagma ebrium | | >25 |
| Insecta | Odonata | | Coenagrionidae | Enallagma exsulans | | <5 |
| Insecta | Odonata | | Coenagrionidae | Enallagma geminatum | | 5to25 |
| Insecta | Odonata | | Coenagrionidae | Enallagma hageni | | >25 |
| Insecta | Odonata | | Coenagrionidae | Enallagma signatum | | 5to25 |
| Insecta | Odonata | | Coenagrionidae | Enallagma traviatum | | lacking |
| Insecta | Odonata | | Coenagrionidae | Enallagma vesperum | | 5to25 |
| Insecta | Odonata | | Coenagrionidae | Ischnura hastata | | 5to25 |
| Insecta | Odonata | | Coenagrionidae | Ischnura kellicotti | | <5 |
| Insecta | Odonata | | Coenagrionidae | Ischnura posita | | 5to25 |
| Insecta | Odonata | | Coenagrionidae | Ischnura verticalis | | 5to25 |
| Insecta | Odonata | | Coenagrionidae | Nehalennia gracilis | | 5to25 |
| Insecta | Odonata | | Coenagrionidae | Nehalennia irene | | >25 |
| Insecta | Odonata | | Cordulegastridae | Cordulegaster bilineata | | lacking |
| Insecta | Odonata | | Cordulegastridae | Cordulegaster diastatops | | 5to25 |
| Insecta | Odonata | | Cordulegastridae | Cordulegaster erronea | | lacking |
| Insecta | Odonata | | Cordulegastridae | Cordulegaster maculata | | >25 |
| Insecta | Odonata | | Cordulegastridae | Cordulegaster obliqua | | <5 |
| Insecta | Odonata | | Corduliidae | Cordulia shurtleffi | | <5 |
| Insecta | Odonata | | Corduliidae | Dorocordulia libera | | 5to25 |
| Insecta | Odonata | | Corduliidae | Epitheca canis | | >25 |
| Insecta | Odonata | | Corduliidae | Epitheca costalis | | lacking |
| Insecta | Odonata | | Corduliidae | Epitheca cynosura | | >25 |
| Insecta | Odonata | | Corduliidae | Epitheca princeps | | <5 |
| Insecta | Odonata | | Corduliidae | Epitheca spinigera | | 5to25 |
| Insecta | Odonata | | Corduliidae | Helocordulia uhleri | | 5to25 |
| Insecta | Odonata | | Corduliidae | Neurocordulia yamaskanensis | | <5 |
| Insecta | Odonata | | Corduliidae | Neurocordulia molesta | | lacking |
| Insecta | Odonata | | Corduliidae | Neurocordulia obsoleta | | lacking |
| Insecta | Odonata | | Corduliidae | Somatochlora cingulata | | 5to25 |
| Insecta | Odonata | | Corduliidae | Somatochlora elongata | | 5to25 |
| Insecta | Odonata | | Corduliidae | Somatochlora ensigera | | lacking |
| Insecta | Odonata | | Corduliidae | Somatochlora forcipata | | <5 |
| Insecta | Odonata | | Corduliidae | Somatochlora franklini | | 5to25 |
| Insecta | Odonata | | Corduliidae | Somatochlora hineana | | lacking |
| Insecta | Odonata | | Corduliidae | Somatochlora incurvata | | <5 |
| Insecta | Odonata | | Corduliidae | Somatochlora kennedyi | | <5 |
| Insecta | Odonata | | Corduliidae | Somatochlora linearis | | lacking |
| Insecta | Odonata | | Corduliidae | Somatochlora minor | | 5to25 |
| Insecta | Odonata | | Corduliidae | Somatochlora tenebrosa | | lacking |
| Insecta | Odonata | | Corduliidae | Somatochlora walshii | | 5to25 |
| Insecta | Odonata | | Corduliidae | Somatochlora williamsoni | | 5to25 |
| Insecta | Odonata | | Corduliidae | Somatochlora albicinta | | 5to25 |
| Insecta | Odonata | | Corduliidae | Somatochlora hudsonica | | 5to25 |
| Insecta | Odonata | | Corduliidae | Williamsonia fletcheri | | lacking |
| Insecta | Odonata | | Corduliidae | Williamsonia lintneri | | lacking |
| Insecta | Odonata | | Gomphidae | Arigomphus cornutus | | lacking |
| Insecta | Odonata | | Gomphidae | Arigomphus furcifer | | lacking |
| Insecta | Odonata | | Gomphidae | Arigomphus submedianus | | lacking |
| Insecta | Odonata | | Gomphidae | Arigomphus villosipes | | <5 |
| Insecta | Odonata | | Gomphidae | Dromogomphus spinosus | | <5 |
| Insecta | Odonata | | Gomphidae | Dromogomphus spoliatus | | lacking |
| Insecta | Odonata | | Gomphidae | Erpetogomphus designatus | | lacking |
| Insecta | Odonata | | Gomphidae | Gomphus borealis | | 5to25 |
| Insecta | Odonata | | Gomphidae | Gomphus descriptus | | 5to25 |
| Insecta | Odonata | | Gomphidae | Gomphus williamsoni | | lacking |
| Insecta | Odonata | | Gomphidae | Gomphus adelphus | | 5to25 |
| Insecta | Odonata | | Gomphidae | Gomphus crassus | | lacking |
| Insecta | Odonata | | Gomphidae | Gomphus exilis | | 5to25 |
| Insecta | Odonata | | Gomphidae | Gomphus fraternus | | lacking |
| Insecta | Odonata | | Gomphidae | Gomphus graslinellus | | 5to25 |
| Insecta | Odonata | | Gomphidae | Gomphus lineatifrons | | lacking |
| Insecta | Odonata | | Gomphidae | Gomphus lividus | | <5 |
| Insecta | Odonata | | Gomphidae | Gomphus quadricolor | | lacking |
| Insecta | Odonata | | Gomphidae | Gomphus spicatus | | >25 |
| Insecta | Odonata | | Gomphidae | Gomphus vastus | | lacking |
| Insecta | Odonata | | Gomphidae | Gomphus ventricosus | | <5 |
| Insecta | Odonata | | Gomphidae | Gomphus viridifrons | | lacking |
| Insecta | Odonata | | Gomphidae | Gompus externus | | lacking |
| Insecta | Odonata | | Gomphidae | Hagenius brevistylus | | 5to25 |
| Insecta | Odonata | | Gomphidae | Lanthus parvulus | | >25 |
| Insecta | Odonata | | Gomphidae | Lanthus vernalis | | zero |
| Insecta | Odonata | | Gomphidae | Ophiogomphus anomalus | | 5to25 |
| Insecta | Odonata | | Gomphidae | Ophiogomphus carolinus | | lacking |
| Insecta | Odonata | | Gomphidae | Ophiogomphus carolus | | 5to25 |
| Insecta | Odonata | | Gomphidae | Ophiogomphus colubrinus | | 5to25 |
| Insecta | Odonata | | Gomphidae | Ophiogomphus howei | | lacking |
| Insecta | Odonata | | Gomphidae | Ophiogomphus mainensis | | >25 |
| Insecta | Odonata | | Gomphidae | Ophiogomphus rupinsulensis | | 5to25 |
| Insecta | Odonata | | Gomphidae | Ophiogomphus smithi | | lacking |
| Insecta | Odonata | | Gomphidae | Ophiogomphus susbehcha | | lacking |
| Insecta | Odonata | | Gomphidae | Progomphus obscurus | | 5to25 |
| Insecta | Odonata | | Gomphidae | Stylogomphus albistylus | | >25 |
| Insecta | Odonata | | Gomphidae | Stylurus amnicola | | lacking |
| Insecta | Odonata | | Gomphidae | Stylurus laurae | | lacking |
| Insecta | Odonata | | Gomphidae | Stylurus notatus | | lacking |
| Insecta | Odonata | | Gomphidae | Stylurus plagiatus | | lacking |
| Insecta | Odonata | | Gomphidae | Stylurus scudderi | | lacking |
| Insecta | Odonata | | Gomphidae | Stylurus spiniceps | | lacking |
| Insecta | Odonata | | Lestidae | Archilestes grandis | | <5 |
| Insecta | Odonata | | Lestidae | Lestes congener | | 5to25 |
| Insecta | Odonata | | Lestidae | Lestes disjunctus | | 5to25 |
| Insecta | Odonata | | Lestidae | Lestes dryas | | 5to25 |
| Insecta | Odonata | | Lestidae | Lestes eurinus | | <5 |
| Insecta | Odonata | | Lestidae | Lestes forcipatus | | 5to25 |
| Insecta | Odonata | | Lestidae | Lestes inaequalis | | <5 |
| Insecta | Odonata | | Lestidae | Lestes rectangularis | | 5to25 |
| Insecta | Odonata | | Lestidae | Lestes unguiculatus | | >25 |
| Insecta | Odonata | | Lestidae | Lestes vigilax | | zero |
| Insecta | Odonata | | Libellulidae | Celithemis elisa | | 5to25 |
| Insecta | Odonata | | Libellulidae | Celithemis eponina | | <5 |
| Insecta | Odonata | | Libellulidae | Celithemis fasciata | | lacking |
| Insecta | Odonata | | Libellulidae | Erythemis simplicicollis | | <5 |
| Insecta | Odonata | | Libellulidae | Erythrodiplax berenice | | zero |
| Insecta | Odonata | | Libellulidae | Ladona julia | | 5to25 |
| Insecta | Odonata | | Libellulidae | Leucorrhinia frigida | | 5to25 |
| Insecta | Odonata | | Libellulidae | Leucorrhinia glacialis | | 5to25 |
| Insecta | Odonata | | Libellulidae | Leucorrhinia hudsonica | | 5to25 |
| Insecta | Odonata | | Libellulidae | Leucorrhinia intacta | | 5to25 |
| Insecta | Odonata | | Libellulidae | Leucorrhinia proxima | | 5to25 |
| Insecta | Odonata | | Libellulidae | Leucorrhinia patricia | | 5to25 |
| Insecta | Odonata | | Libellulidae | Libellula auripennis | | <5 |
| Insecta | Odonata | | Libellulidae | Libellula cyanea | | lacking |
| Insecta | Odonata | | Libellulidae | Libellula incesta | | <5 |
| Insecta | Odonata | | Libellulidae | Libellula luctuosa | | 5to25 |
| Insecta | Odonata | | Libellulidae | Libellula pulchella | | 5to25 |
| Insecta | Odonata | | Libellulidae | Libellula quadrimaculata | | >25 |
| Insecta | Odonata | | Libellulidae | Libellula semifasciata | | <5 |
| Insecta | Odonata | | Libellulidae | Libellula vibrans | | 5to25 |
| Insecta | Odonata | | Libellulidae | Nannothemis bella | | lacking |
| Insecta | Odonata | | Libellulidae | Pachydiplax longipennis | | 5to25 |
| Insecta | Odonata | | Libellulidae | Pantala flavescens | | 5to25 |
| Insecta | Odonata | | Libellulidae | Pantala hymenaea | | zero |
| Insecta | Odonata | | Libellulidae | Perithemis tenera | | <5 |
| Insecta | Odonata | | Libellulidae | Plathemis lydia | | >25 |
| Insecta | Odonata | | Libellulidae | Sympetrum ambiguum | | <5 |
| Insecta | Odonata | | Libellulidae | Sympetrum corruptum | | 5to25 |
| Insecta | Odonata | | Libellulidae | Sympetrum costiferum | | 5to25 |
| Insecta | Odonata | | Libellulidae | Sympetrum danae | | >25 |
| Insecta | Odonata | | Libellulidae | Sympetrum internum | | >25 |
| Insecta | Odonata | | Libellulidae | Sympetrum janeae | | >25 |
| Insecta | Odonata | | Libellulidae | Sympetrum obtrusum | | >25 |
| Insecta | Odonata | | Libellulidae | Sympetrum occidentale | | 5to25 |
| Insecta | Odonata | | Libellulidae | Sympetrum rubicundulum | | 5to25 |
| Insecta | Odonata | | Libellulidae | Sympetrum semicinctum | | 5to25 |
| Insecta | Odonata | | Libellulidae | Sympetrum vicinum | | 5to25 |
| Insecta | Odonata | | Libellulidae | Tramea carolina | | <5 |
| Insecta | Odonata | | Libellulidae | Tramea lacerata | | 5to25 |
| Insecta | Odonata | | Libellulidae | Tramea onusta | | zero |
| Insecta | Odonata | | Macromiidae | Didymops transversa | | 5to25 |
| Insecta | Odonata | | Macromiidae | Macromia illinoiensis | | 5to25 |
| Insecta | Odonata | | Macromiidae | Macromia taeniolata | | <5 |
| Insecta | Odonata | | Macromiidae | Macromia pacifica | | lacking |
| Insecta | Odonata | | Petaluridae | Tachopteryx thoreyi | | zero |
| Insecta | Plecoptera | | Capniidae | Paracapnia angulata | | 5to25 |
| Insecta | Plecoptera | | Chloroperlidae | Haploperla brevis | | >25 |
| Insecta | Plecoptera | | Leuctridae | Leuctra tenuis | | 5to25 |
| Insecta | Plecoptera | | Nemouridae | Nemoura trispinosa | | 5to25 |
| Insecta | Plecoptera | | Perlidae | Acroneuria lycorias | | 5to25 |
| Insecta | Plecoptera | | Perlidae | Paragnetina media | | 5to25 |
| Insecta | Plecoptera | | Perlodidae | Arcynopteryx compacta | | >25 |
| Insecta | Plecoptera | | Perlodidae | Isogenoides frontalis | | <5 |
| Insecta | Plecoptera | | Perlodidae | Isoperla bilineata | | >25 |
| Insecta | Trichoptera | | Brachycentridae | Brachycentrus americanus | | >25 |
| Insecta | Trichoptera | | Brachycentridae | Brachycentrus lateralis | | <5 |
| Insecta | Trichoptera | | Brachycentridae | Molanna ulmerina | | 5to25 |
| Insecta | Trichoptera | | Dipseudopsidae | Phylocentropus placidus | | >25 |
| Insecta | Trichoptera | | Helicopsychidae | Helicopsyche borealis | | >25 |
| Insecta | Trichoptera | | Hydropsychidae | Ceratopsyche bronta | | lacking |
| Insecta | Trichoptera | | Hydropsychidae | Ceratopsyche slossonae | | lacking |
| Insecta | Trichoptera | | Hydropsychidae | Cheumatopsyche burksi | | <5 |
| Insecta | Trichoptera | | Hydropsychidae | Cheumatopsyche campyla | | >25 |
| Insecta | Trichoptera | | Hydropsychidae | Cheumatopsyche minuscula | | 5to25 |
| Insecta | Trichoptera | | Hydropsychidae | Cheumatopsyche pasella | | 5to25 |
| Insecta | Trichoptera | | Hydropsychidae | Cheumatopsyche speciosa | | 5to25 |
| Insecta | Trichoptera | | Hydropsychidae | Hydropsyche alternans | | >25 |
| Insecta | Trichoptera | | Hydropsychidae | Hydropsyche betteni | | >25 |
| Insecta | Trichoptera | | Hydropsychidae | Hydropsyche guttata | | 5to25 |
| Insecta | Trichoptera | | Hydropsychidae | Hydropsyche hageni | | 5to25 |
| Insecta | Trichoptera | | Hydropsychidae | Hydropsyche incommoda | | <5 |
| Insecta | Trichoptera | | Hydropsychidae | Hydropsyche phalerata | | 5to25 |
| Insecta | Trichoptera | | Hydropsychidae | Hydropsyche recurvata | | lacking |
| Insecta | Trichoptera | | Hydropsychidae | Hydropsyche valanis | | lacking |
| Insecta | Trichoptera | | Hydropsychidae | Hydropsyche walkeri | | 5to25 |
| Insecta | Trichoptera | | Hydropsychidae | Hydropsyche morosa | | >25 |
| Insecta | Trichoptera | | Hydropsychidae | Macrostemum zebratum | | 5to25 |
| Insecta | Trichoptera | | Hydropsychidae | Potamyia flava | | >25 |
| Insecta | Trichoptera | | Hydroptilidae | Agraylea multipunctata | | >25 |
| Insecta | Trichoptera | | Hydroptilidae | Hydroptila armata | | >25 |
| Insecta | Trichoptera | | Hydroptilidae | Hydroptila consimilis | | >25 |
| Insecta | Trichoptera | | Hydroptilidae | Hydroptila perdita | | 5to25 |
| Insecta | Trichoptera | | Hydroptilidae | Hydroptila spatulata | | 5to25 |
| Insecta | Trichoptera | | Hydroptilidae | Hydroptila waubesiana | | >25 |
| Insecta | Trichoptera | | Hydroptilidae | Ithytrichia clavata | | <5 |
| Insecta | Trichoptera | | Hydroptilidae | Leucotrichia pictipes | | >25 |
| Insecta | Trichoptera | | Hydroptilidae | Neotrichia halia | | lacking |
| Insecta | Trichoptera | | Hydroptilidae | Ochrotrichia tarsalis | | 5to25 |
| Insecta | Trichoptera | | Hydroptilidae | Orthotrichia aegerfasciella | | <5 |
| Insecta | Trichoptera | | Hydroptilidae | Orthotrichia cristata | | 5to25 |
| Insecta | Trichoptera | | Hydroptilidae | Oxythira pallida | | <5 |
| Insecta | Trichoptera | | Hydroptilidae | Oxythira verna | | <5 |
| Insecta | Trichoptera | | Lepidostomatidae | Lepidostoma togatum | | >25 |
| Insecta | Trichoptera | | Leptoceridae | Ceraclea alagma | | >25 |
| Insecta | Trichoptera | | Leptoceridae | Ceraclea ancylus | | 5to25 |
| Insecta | Trichoptera | | Leptoceridae | Ceraclea angusta | | lacking |
| Insecta | Trichoptera | | Leptoceridae | Ceraclea annulicornis | | >25 |
| Insecta | Trichoptera | | Leptoceridae | Ceraclea cancellata | | >25 |
| Insecta | Trichoptera | | Leptoceridae | Ceraclea diluta | | 5to25 |
| Insecta | Trichoptera | | Leptoceridae | Ceraclea erratica | | lacking |
| Insecta | Trichoptera | | Leptoceridae | Ceraclea flava | | 5to25 |
| Insecta | Trichoptera | | Leptoceridae | Ceraclea mentiea | | <5 |
| Insecta | Trichoptera | | Leptoceridae | Ceraclea neffi | | <5 |
| Insecta | Trichoptera | | Leptoceridae | Ceraclea nepha | | 5to25 |
| Insecta | Trichoptera | | Leptoceridae | Ceraclea resurgens | | >25 |
| Insecta | Trichoptera | | Leptoceridae | Ceraclea submacula | | lacking |
| Insecta | Trichoptera | | Leptoceridae | Ceraclea tarsipunctata | | >25 |
| Insecta | Trichoptera | | Leptoceridae | Ceraclea transversa | | >25 |
| Insecta | Trichoptera | | Leptoceridae | Leptocerus interruptus | | <5 |
| Insecta | Trichoptera | | Leptoceridae | Mystacides interjecta  (in BOLD as M. interjectus) | | >25 |
| Insecta | Trichoptera | | Leptoceridae | Mystacides longicornis | | 5to25 |
| Insecta | Trichoptera | | Leptoceridae | Mystacides sepulchralis | | >25 |
| Insecta | Trichoptera | | Leptoceridae | Nectopsyche diarina | | 5to25 |
| Insecta | Trichoptera | | Leptoceridae | Nectopsyche exquisita | | 5to25 |
| Insecta | Trichoptera | | Leptoceridae | Oecetis avara | | >25 |
| Insecta | Trichoptera | | Leptoceridae | Oecetis cinerascens | | >25 |
| Insecta | Trichoptera | | Leptoceridae | Oecetis eddlestoni | | <5 |
| Insecta | Trichoptera | | Leptoceridae | Oecetis immobilis | | 5to25 |
| Insecta | Trichoptera | | Leptoceridae | Oecetis inconspicua | | >25 |
| Insecta | Trichoptera | | Leptoceridae | Oecetis osteni | | 5to25 |
| Insecta | Trichoptera | | Leptoceridae | Setodes incertus | | 5to25 |
| Insecta | Trichoptera | | Leptoceridae | Setodes oligius | | <5 |
| Insecta | Trichoptera | | Leptoceridae | Triaenodes flavescens | | 5to25 |
| Insecta | Trichoptera | | Leptoceridae | Triaenodes frontalis | | >25 |
| Insecta | Trichoptera | | Leptoceridae | Triaenodes injusta  (in BOLD as T. injustus) | | 5to25 |
| Insecta | Trichoptera | | Leptoceridae | Triaenodes tardus | | >25 |
| Insecta | Trichoptera | | Limnephilidae | Arctopora pulchella | | 5to25 |
| Insecta | Trichoptera | | Limnephilidae | Arctopora salmon | | <5 |
| Insecta | Trichoptera | | Limnephilidae | Glyphopsyche missouri | | lacking |
| Insecta | Trichoptera | | Limnephilidae | Glyphopsyche sequatchie | | <5 |
| Insecta | Trichoptera | | Limnephilidae | Limnephilus hyalinus | | 5to25 |
| Insecta | Trichoptera | | Limnephilidae | Limnephilus indivisus | | >25 |
| Insecta | Trichoptera | | Limnephilidae | Limnephilus infernalis | | >25 |
| Insecta | Trichoptera | | Limnephilidae | Limnephilus sackeni | | lacking |
| Insecta | Trichoptera | | Limnephilidae | Limnephilus submonilifer | | >25 |
| Insecta | Trichoptera | | Limnephilidae | Nemotaulius hostilis | | 5to25 |
| Insecta | Trichoptera | | Limnephilidae | Psychoglypha subborealis | | >25 |
| Insecta | Trichoptera | | Molannidae | Molanna flavicornis | | >25 |
| Insecta | Trichoptera | | Molannidae | Molanna uniophila | | >25 |
| Insecta | Trichoptera | | Philopotamoidea | Chimarra obscura | | >25 |
| Insecta | Trichoptera | | Phryganeidae | Agrypnia straminea | | >25 |
| Insecta | Trichoptera | | Phryganeidae | Agrypnia vestita | | 5to25 |
| Insecta | Trichoptera | | Phryganeidae | Banksiola crotchi | | >25 |
| Insecta | Trichoptera | | Phryganeidae | Fabria inornata | | 5to25 |
| Insecta | Trichoptera | | Phryganeidae | Phryganea cinerea | | >25 |
| Insecta | Trichoptera | | Polycentropodidae | Cernotina ohio | | lacking |
| Insecta | Trichoptera | | Polycentropodidae | Cyrnellus fraternus | | >25 |
| Insecta | Trichoptera | | Polycentropodidae | Neureclipsis bimaculata | | 5to25 |
| Insecta | Trichoptera | | Polycentropodidae | Neureclipsis crepuscularis | | >25 |
| Insecta | Trichoptera | | Polycentropodidae | Neureclipsis valida | | 5to25 |
| Insecta | Trichoptera | | Polycentropodidae | Nyctiophylax affinis | | 5to25 |
| Insecta | Trichoptera | | Polycentropodidae | Nyctiophylax moestus | | 5to25 |
| Insecta | Trichoptera | | Polycentropodidae | Nyctiophylax visitus | | lacking |
| Insecta | Trichoptera | | Polycentropodidae | Polycentropus centralis | | 5to25 |
| Insecta | Trichoptera | | Polycentropodidae | Polycentropus cinereus | | >25 |
| Insecta | Trichoptera | | Polycentropodidae | Polycentropus crassicornis | | <5 |
| Insecta | Trichoptera | | Polycentropodidae | Polycentropus interruptus | | zero |
| Insecta | Trichoptera | | Polycentropodidae | Polycentropus remotus | | zero |
| Insecta | Trichoptera | | Psycomyiidae | Lype diversa | | >25 |
| Insecta | Trichoptera | | Psycomyiidae | Psychomyia flavida | | >25 |
| Insecta | Trichoptera | | Rhyacophilidae | Rhyacophila atrata | | 5to25 |
| **Phylum *Bryozoa*** |  | |  |  | |  |
| Gymnolaemata | Ctenostomata | | Paludicellidae | Paludicella articulata | | lacking |
| Gymnolaemata | Ctenostomata | | Paludicellidae | Pottsiella erecta | | lacking |
| Phylactolaemata | Plumatellida | | Cristatellidae | Cristatella mucedo | | <5 |
| Phylactolaemata | Plumatellida | | Lophopodidae | **Lophopodella carteri** | | lacking |
| Phylactolaemata | Plumatellida | | Pectinatellidae | Pectinatella magnifica | | <5 |
| Phylactolaemata | Plumatellida | | Plumatellidae | Plumatella casminana | | lacking |
| Phylactolaemata | Plumatellida | | Plumatellidae | Plumatella emarginata | | lacking |
| Phylactolaemata | Plumatellida | | Plumatellidae | Plumatella repens | | <5 |
| **Phylum *Chordata*** |  | |  |  | |  |
| Actinopterygii | Acipenseriformes | | Acipenseridae | *Acipenser fulvescens* | | 5to25 |
| Actinopterygii | Acipenseriformes | | Polyodontidae | *Polyodon spathula* | | 5to25 |
| Actinopterygii | Amiiformes | | Amiidae | *Amia calva* | | 5to25 |
| Actinopterygii | Anguilliformes | | Anguillidae | *Anguilla rostrata* | | >25 |
| Actinopterygii | Atheriniformes | | Atherinopsidae | *Labidesthes sicculus* | | >25 |
| Actinopterygii | Clupeiformes | | Clupeidae | ***Alosa aestivalis*** | | 5to25 |
| Actinopterygii | Clupeiformes | | Clupeidae | ***Alosa chrysochloris*** | | <5 |
| Actinopterygii | Clupeiformes | | Clupeidae | ***Alosa pseudoharengus*** | | >25 |
| Actinopterygii | Clupeiformes | | Clupeidae | ***Alosa sapidissima*** | | >25 |
| Actinopterygii | Clupeiformes | | Clupeidae | ***Dorosoma cepedianum*** | | >25 |
| Actinopterygii | Cypriniformes | | Catostomidae | *Carpiodes carpio* | | 5to25 |
| Actinopterygii | Cypriniformes | | Catostomidae | *Carpiodes cyprinus* | | >25 |
| Actinopterygii | Cypriniformes | | Catostomidae | *Catostomus catostomus* | | >25 |
| Actinopterygii | Cypriniformes | | Catostomidae | *Catostomus commersonii* | | >25 |
| Actinopterygii | Cypriniformes | | Catostomidae | *Erimyzon oblongus* | | 5to25 |
| Actinopterygii | Cypriniformes | | Catostomidae | *Erimyzon sucetta* | | 5to25 |
| Actinopterygii | Cypriniformes | | Catostomidae | *Hypentelium nigricans* | | >25 |
| Actinopterygii | Cypriniformes | | Catostomidae | *Ictiobus cyprinellus* | | 5to25 |
| Actinopterygii | Cypriniformes | | Catostomidae | *Ictiobus niger* | | 5to25 |
| Actinopterygii | Cypriniformes | | Catostomidae | *Minytrema melanops* | | 5to25 |
| Actinopterygii | Cypriniformes | | Catostomidae | *Moxostoma anisurum* | | >25 |
| Actinopterygii | Cypriniformes | | Catostomidae | *Moxostoma carinatum* | | 5to25 |
| Actinopterygii | Cypriniformes | | Catostomidae | *Moxostoma duquesnii* | | 5to25 |
| Actinopterygii | Cypriniformes | | Catostomidae | *Moxostoma erythrurum* | | >25 |
| Actinopterygii | Cypriniformes | | Catostomidae | *Moxostoma macrolepidotum* | | >25 |
| Actinopterygii | Cypriniformes | | Catostomidae | *Moxostoma valenciennesi* | | 5to25 |
| Actinopterygii | Cypriniformes | | Cobitidae | ***Misgurnus anguillicaudatus*** | | 5to25 |
| Actinopterygii | Cypriniformes | | Cyprinidae | *Campostoma anomalum* | | >25 |
| Actinopterygii | Cypriniformes | | Cyprinidae | *Campostoma oligolepis* | | 5to25 |
| Actinopterygii | Cypriniformes | | Cyprinidae | ***Carassius auratus*** | | >25 |
| Actinopterygii | Cypriniformes | | Cyprinidae | *Chrosomus eos* | | >25 |
| Actinopterygii | Cypriniformes | | Cyprinidae | *Chrosomus erythrogaster* | | 5to25 |
| Actinopterygii | Cypriniformes | | Cyprinidae | *Chrosomus neogaeus* | | >25 |
| Actinopterygii | Cypriniformes | | Cyprinidae | *Clinostomus elongatus* | | 5to25 |
| Actinopterygii | Cypriniformes | | Cyprinidae | *Couesius plumbeus* | | 5to25 |
| Actinopterygii | Cypriniformes | | Cyprinidae | ***Ctenopharyngodon idella*** | | >25 |
| Actinopterygii | Cypriniformes | | Cyprinidae | *Cyprinella analostana* | | >25 |
| Actinopterygii | Cypriniformes | | Cyprinidae | ***Cyprinella lutrensis*** | | 5to25 |
| Actinopterygii | Cypriniformes | | Cyprinidae | *Cyprinella spiloptera* | | >25 |
| Actinopterygii | Cypriniformes | | Cyprinidae | ***Cyprinus carpio*** | | >25 |
| Actinopterygii | Cypriniformes | | Cyprinidae | *Exoglossum laurae* | | <5 |
| Actinopterygii | Cypriniformes | | Cyprinidae | *Exoglossum maxillingua* | | 5to25 |
| Actinopterygii | Cypriniformes | | Cyprinidae | *Hybognathus hankinsoni* | | 5to25 |
| Actinopterygii | Cypriniformes | | Cyprinidae | *Hybognathus regius* | | 5to25 |
| Actinopterygii | Cypriniformes | | Cyprinidae | *Hybopsis amblops* | | 5to25 |
| Actinopterygii | Cypriniformes | | Cyprinidae | *Luxilus chrysocephalus* | | >25 |
| Actinopterygii | Cypriniformes | | Cyprinidae | *Luxilus cornutus* | | >25 |
| Actinopterygii | Cypriniformes | | Cyprinidae | *Lythrurus umbratilis* | | 5to25 |
| Actinopterygii | Cypriniformes | | Cyprinidae | *Macrhybopsis storeriana* | | 5to25 |
| Actinopterygii | Cypriniformes | | Cyprinidae | *Margariscus margarita* | | >25 |
| Actinopterygii | Cypriniformes | | Cyprinidae | *Nocomis biguttatus* | | 5to25 |
| Actinopterygii | Cypriniformes | | Cyprinidae | *Nocomis micropogon* | | 5to25 |
| Actinopterygii | Cypriniformes | | Cyprinidae | *Notemigonus crysoleucas* | | >25 |
| Actinopterygii | Cypriniformes | | Cyprinidae | *Notropis anogenus* | | 5to25 |
| Actinopterygii | Cypriniformes | | Cyprinidae | *Notropis atherinoides* | | >25 |
| Actinopterygii | Cypriniformes | | Cyprinidae | *Notropis bifrenatus* | | >25 |
| Actinopterygii | Cypriniformes | | Cyprinidae | *Notropis blennius* | | 5to25 |
| Actinopterygii | Cypriniformes | | Cyprinidae | *Notropis boops* | | 5to25 |
| Actinopterygii | Cypriniformes | | Cyprinidae | *Notropis buccatus*  (in BOLD as *Ericymba buccata*) | | 5to25 |
| Actinopterygii | Cypriniformes | | Cyprinidae | ***Notropis buchanani*** | | >25 |
| Actinopterygii | Cypriniformes | | Cyprinidae | *Notropis chalybaeus* | | 5to25 |
| Actinopterygii | Cypriniformes | | Cyprinidae | *Notropis dorsalis* | | 5to25 |
| Actinopterygii | Cypriniformes | | Cyprinidae | *Notropis heterodon* | | 5to25 |
| Actinopterygii | Cypriniformes | | Cyprinidae | *Notropis heterolepis* | | 5to25 |
| Actinopterygii | Cypriniformes | | Cyprinidae | *Notropis hudsonius* | | >25 |
| Actinopterygii | Cypriniformes | | Cyprinidae | *Notropis photogenis* | | >25 |
| Actinopterygii | Cypriniformes | | Cyprinidae | *Notropis procne* | | 5to25 |
| Actinopterygii | Cypriniformes | | Cyprinidae | *Notropis rubellus* | | >25 |
| Actinopterygii | Cypriniformes | | Cyprinidae | *Notropis stramineus* | | >25 |
| Actinopterygii | Cypriniformes | | Cyprinidae | *Notropis volucellus* | | >25 |
| Actinopterygii | Cypriniformes | | Cyprinidae | *Opsopoeodus emiliae* | | <5 |
| Actinopterygii | Cypriniformes | | Cyprinidae | ***Phenacobius mirabilis*** | | 5to25 |
| Actinopterygii | Cypriniformes | | Cyprinidae | *Pimephales notatus* | | >25 |
| Actinopterygii | Cypriniformes | | Cyprinidae | *Pimephales promelas* | | >25 |
| Actinopterygii | Cypriniformes | | Cyprinidae | ***Pimephales vigilax*** | | 5to25 |
| Actinopterygii | Cypriniformes | | Cyprinidae | *Rhinichthys atratulus* | | >25 |
| Actinopterygii | Cypriniformes | | Cyprinidae | *Rhinichthys cataractae* | | >25 |
| Actinopterygii | Cypriniformes | | Cyprinidae | *Rhinichthys obtusus* | | >25 |
| Actinopterygii | Cypriniformes | | Cyprinidae | ***Scardinius erythrophthalmus*** | | >25 |
| Actinopterygii | Cypriniformes | | Cyprinidae | *Semotilus atromaculatus* | | >25 |
| Actinopterygii | Cypriniformes | | Cyprinidae | *Semotilus corporalis* | | >25 |
| Actinopterygii | Cyprinodontiformes | | Fundulidae | *Fundulus diaphanus* | | >25 |
| Actinopterygii | Cyprinodontiformes | | Fundulidae | *Fundulus dispar* | | <5 |
| Actinopterygii | Cyprinodontiformes | | Fundulidae | *Fundulus notatus* | | 5to25 |
| Actinopterygii | Cyprinodontiformes | | Poeciliidae | ***Gambusia affinis*** | | >25 |
| Actinopterygii | Esociformes | | Esocidae | *Esox americanus* | | >25 |
| Actinopterygii | Esociformes | | Esocidae | *Esox lucius* | | >25 |
| Actinopterygii | Esociformes | | Esocidae | *Esox masquinongy* | | >25 |
| Actinopterygii | Esociformes | | Esocidae | ***Esox niger*** | | >25 |
| Actinopterygii | Esociformes | | Umbridae | *Umbra limi* | | >25 |
| Actinopterygii | Gadiformes | | Gadidae | *Lota lota* | | >25 |
| Actinopterygii | Gasterosteiformes | | Gasterosteidae | ***Apeltes quadracus*** | | 5to25 |
| Actinopterygii | Gasterosteiformes | | Gasterosteidae | *Culaea inconstans* | | >25 |
| Actinopterygii | Gasterosteiformes | | Gasterosteidae | ***Gasterosteus aculeatus*** | | >25 |
| Actinopterygii | Gasterosteiformes | | Gasterosteidae | *Pungitius pungitius* | | >25 |
| Actinopterygii | Osmeriformes | | Osmeridae | ***Osmerus mordax*** | | >25 |
| Actinopterygii | Osteoglossiformes | | Hiodontidae | *Hiodon tergisus* | | 5to25 |
| Actinopterygii | Perciformes | | Centrarchidae | *Ambloplites rupestris* | | >25 |
| Actinopterygii | Perciformes | | Centrarchidae | *Chaenobryttus gulosus* | | 5to25 |
| Actinopterygii | Perciformes | | Centrarchidae | ***Enneacanthus gloriosus*** | | >25 |
| Actinopterygii | Perciformes | | Centrarchidae | *Lepomis cyanellus* | | >25 |
| Actinopterygii | Perciformes | | Centrarchidae | *Lepomis gibbosus* | | >25 |
| Actinopterygii | Perciformes | | Centrarchidae | *Lepomis gulosus* | | 5to25 |
| Actinopterygii | Perciformes | | Centrarchidae | ***Lepomis humilis*** | | 5to25 |
| Actinopterygii | Perciformes | | Centrarchidae | *Lepomis macrochirus* | | >25 |
| Actinopterygii | Perciformes | | Centrarchidae | *Lepomis megalotis* | | >25 |
| Actinopterygii | Perciformes | | Centrarchidae | ***Lepomis microlophus*** | | 5to25 |
| Actinopterygii | Perciformes | | Centrarchidae | *Micropterus dolomieu* | | >25 |
| Actinopterygii | Perciformes | | Centrarchidae | *Micropterus salmoides* | | >25 |
| Actinopterygii | Perciformes | | Centrarchidae | *Pomoxis annularis* | | 5to25 |
| Actinopterygii | Perciformes | | Centrarchidae | *Pomoxis nigromaculatus* | | 5to25 |
| Actinopterygii | Perciformes | | Gobiidae | ***Neogobius melanostomus*** | | >25 |
| Actinopterygii | Perciformes | | Gobiidae | ***Proterorhinus marmoratus*** | | 5to25 |
| Actinopterygii | Perciformes | | Moronidae | ***Morone americana*** | | >25 |
| Actinopterygii | Perciformes | | Moronidae | *Morone chrysops* | | >25 |
| Actinopterygii | Perciformes | | Moronidae | *Morone mississippiensis* | | 5to25 |
| Actinopterygii | Perciformes | | Percidae | *Ammocrypta clara* | | 5to25 |
| Actinopterygii | Perciformes | | Percidae | *Ammocrypta pellucida* | | >25 |
| Actinopterygii | Perciformes | | Percidae | *Etheostoma blennioides* | | >25 |
| Actinopterygii | Perciformes | | Percidae | *Etheostoma caeruleum* | | >25 |
| Actinopterygii | Perciformes | | Percidae | *Etheostoma chlorosomum* | | 5to25 |
| Actinopterygii | Perciformes | | Percidae | *Etheostoma exile* | | >25 |
| Actinopterygii | Perciformes | | Percidae | *Etheostoma flabellare* | | >25 |
| Actinopterygii | Perciformes | | Percidae | *Etheostoma microperca* | | >25 |
| Actinopterygii | Perciformes | | Percidae | *Etheostoma nigrum* | | >25 |
| Actinopterygii | Perciformes | | Percidae | *Etheostoma olmstedi* | | >25 |
| Actinopterygii | Perciformes | | Percidae | *Etheostoma spectabile* | | >25 |
| Actinopterygii | Perciformes | | Percidae | *Etheostoma zonale* | | >25 |
| Actinopterygii | Perciformes | | Percidae | ***Gymnocephalus cernuus*** | | >25 |
| Actinopterygii | Perciformes | | Percidae | *Perca flavescens* | | >25 |
| Actinopterygii | Perciformes | | Percidae | *Percina caprodes* | | >25 |
| Actinopterygii | Perciformes | | Percidae | *Percina copelandi* | | 5to25 |
| Actinopterygii | Perciformes | | Percidae | *Percina evides* | | 5to25 |
| Actinopterygii | Perciformes | | Percidae | *Percina maculata* | | 5to25 |
| Actinopterygii | Perciformes | | Percidae | *Percina phoxocephala* | | 5to25 |
| Actinopterygii | Perciformes | | Percidae | *Percina shumardi* | | 5to25 |
| Actinopterygii | Perciformes | | Percidae | *Sander canadensis* | | 5to25 |
| Actinopterygii | Perciformes | | Percidae | *Sander vitreus* | | >25 |
| Actinopterygii | Perciformes | | Sciaenidae | *Aplodinotus grunniens* | | 5to25 |
| Actinopterygii | Percopsiformes | | Aphredoderidae | *Aphredoderus sayanus* | | 5to25 |
| Actinopterygii | Percopsiformes | | Percopsidae | *Percopsis omiscomaycus* | | >25 |
| Actinopterygii | Salmoniformes | | Salmonidae | *Coregonus artedi* | | >25 |
| Actinopterygii | Salmoniformes | | Salmonidae | *Coregonus clupeaformis* | | >25 |
| Actinopterygii | Salmoniformes | | Salmonidae | *Coregonus hoyi* | | >25 |
| Actinopterygii | Salmoniformes | | Salmonidae | *Coregonus kiyi* | | <5 |
| Actinopterygii | Salmoniformes | | Salmonidae | *Coregonus nigripinnis* | | <5 |
| Actinopterygii | Salmoniformes | | Salmonidae | *Coregonus reighardi* | | Lacking |
| Actinopterygii | Salmoniformes | | Salmonidae | *Coregonus zenithicus* | | <5 |
| Actinopterygii | Salmoniformes | | Salmonidae | ***Oncorhynchus gorbuscha*** | | >25 |
| Actinopterygii | Salmoniformes | | Salmonidae | ***Oncorhynchus kisutch*** | | >25 |
| Actinopterygii | Salmoniformes | | Salmonidae | ***Oncorhynchus mykiss*** | | >25 |
| Actinopterygii | Salmoniformes | | Salmonidae | ***Oncorhynchus nerka*** | | >25 |
| Actinopterygii | Salmoniformes | | Salmonidae | ***Oncorhynchus tshawytscha*** | | >25 |
| Actinopterygii | Salmoniformes | | Salmonidae | *Prosopium coulterii* | | 5to25 |
| Actinopterygii | Salmoniformes | | Salmonidae | *Prosopium cylindraceum* | | >25 |
| Actinopterygii | Salmoniformes | | Salmonidae | *Salmo salar* | | >25 |
| Actinopterygii | Salmoniformes | | Salmonidae | ***Salmo trutta*** | | >25 |
| Actinopterygii | Salmoniformes | | Salmonidae | *Salvelinus fontinalis* | | >25 |
| Actinopterygii | Salmoniformes | | Salmonidae | *Salvelinus namaycush* | | >25 |
| Actinopterygii | Salmoniformes | | Salmonidae | *Thymallus arcticus* | | >25 |
| Actinopterygii | Scorpaeniformes | | Cottidae | *Cottus bairdii* | | >25 |
| Actinopterygii | Scorpaeniformes | | Cottidae | *Cottus cognatus* | | >25 |
| Actinopterygii | Scorpaeniformes | | Cottidae | *Cottus ricei* | | 5to25 |
| Actinopterygii | Scorpaeniformes | | Cottidae | *Myoxocephalus thompsonii* | | 5to25 |
| Actinopterygii | Semionotiformes | | Lepisosteidae | *Lepisosteus oculatus* | | 5to25 |
| Actinopterygii | Semionotiformes | | Lepisosteidae | *Lepisosteus osseus* | | 5to25 |
| Actinopterygii | Semionotiformes | | Lepisosteidae | ***Lepisosteus platostomus*** | | 5to25 |
| Actinopterygii | Siluriformes | | Ictaluridae | ***Ameiurus catus*** | | 5to25 |
| Actinopterygii | Siluriformes | | Ictaluridae | *Ameiurus melas* | | 5to25 |
| Actinopterygii | Siluriformes | | Ictaluridae | *Ameiurus natalis* | | 5to25 |
| Actinopterygii | Siluriformes | | Ictaluridae | *Ameiurus nebulosus* | | >25 |
| Actinopterygii | Siluriformes | | Ictaluridae | *Ictalurus punctatus* | | >25 |
| Actinopterygii | Siluriformes | | Ictaluridae | *Noturus flavus* | | 5to25 |
| Actinopterygii | Siluriformes | | Ictaluridae | *Noturus gyrinus* | | >25 |
| Actinopterygii | Siluriformes | | Ictaluridae | ***Noturus insignis*** | | 5to25 |
| Actinopterygii | Siluriformes | | Ictaluridae | *Noturus miurus* | | 5to25 |
| Actinopterygii | Siluriformes | | Ictaluridae | *Noturus stigmosus* | | <5 |
| Actinopterygii | Siluriformes | | Ictaluridae | *Pylodictis olivaris* | | 5to25 |
| Amphibia | Anura | | Bufonidae | Anaxyrus americanus  (in BOLD as *Bufo americanus*) | | >25 |
| Amphibia | Anura | | Bufonidae | Anaxyrus fowleri  (in BOLD as *Bufo fowleri*) | | >25 |
| Amphibia | Anura | | Hylidae | Acris crepitans | | zero |
| Amphibia | Anura | | Hylidae | Hyla chrysoscelis | | <5 |
| Amphibia | Anura | | Hylidae | Hyla versicolor | | 5to25 |
| Amphibia | Anura | | Hylidae | Pseudacris crucifer | | 5to25 |
| Amphibia | Anura | | Hylidae | Pseudacris maculata | | <5 |
| Amphibia | Anura | | Hylidae | Pseudacris triserita | | 5to25 |
| Amphibia | Anura | | Ranidae | Lithobates blairi | | <5 |
| Amphibia | Anura | | Ranidae | Lithobates catesbeianus  (in BOLD as *Rana catesbeianus*) | | 5to25 |
| Amphibia | Anura | | Ranidae | Lithobates clamitans  (in BOLD as *Rana clamitans*) | | >25 |
| Amphibia | Anura | | Ranidae | Lithobates palustris  (in BOLD as *Rana palustris*) | | 5to25 |
| Amphibia | Anura | | Ranidae | Lithobates pipiens  (in BOLD as *Rana pipiens*) | | 5to25 |
| Amphibia | Anura | | Ranidae | Lithobates septentrionalis  (in BOLD as *Rana septentrionalis*) | | >25 |
| Amphibia | Anura | | Ranidae | Lithobates sylvaticus  (in BOLD as *Rana sylvaticus*) | | <5 |
| Amphibia | Caudata | | Ambystomatidae | Ambystoma jeffersonianum  (in BOLD as *A. jeffersonianum* complex) | | 5to25 |
| Amphibia | Caudata | | Ambystomatidae | Ambystoma laterale | | 5to25 |
| Amphibia | Caudata | | Ambystomatidae | Ambystoma maculatum | | 5to25 |
| Amphibia | Caudata | | Ambystomatidae | Ambystoma opacum | | 5to25 |
| Amphibia | Caudata | | Ambystomatidae | Ambystoma texanum | | 5to25 |
| Amphibia | Caudata | | Ambystomatidae | Ambystoma tigrinum | | 5to25 |
| Amphibia | Caudata | | Cryptobranchidae | Cryptobranchus alleganiensis | | 5to25 |
| Amphibia | Caudata | | Plethodontidae | Desmognathus fuscus | | >25 |
| Amphibia | Caudata | | Plethodontidae | Desmognathus ochrophaeus | | 5to25 |
| Amphibia | Caudata | | Plethodontidae | Eurycea bislineata | | >25 |
| Amphibia | Caudata | | Plethodontidae | Eurycea longicauda | | zero |
| Amphibia | Caudata | | Plethodontidae | Gyrinophilus porphyriticus | | <5 |
| Amphibia | Caudata | | Plethodontidae | Hemidactylium scutatum | | >25 |
| Amphibia | Caudata | | Plethodontidae | Plethodon cinereus | | >25 |
| Amphibia | Caudata | | Plethodontidae | Plethodon glutinosus | | <5 |
| Amphibia | Caudata | | Plethodontidae | Plethodon richmondi | | zero |
| Amphibia | Caudata | | Plethodontidae | Pseudotriton ruber | | <5 |
| Amphibia | Caudata | | Proteidae | Necturus maculosus | | 5to25 |
| Amphibia | Caudata | | Salamandridae | Notophthalmus viridescens | | <5 |
| Amphibia | Caudata | | Sirenidae | Siren intermedia | | <5 |
| Cephalaspidomorphi | Petromyzontiformes | | Petromyzontidae | *Ichthyomyzon castaneus* | | 5to25 |
| Cephalaspidomorphi | Petromyzontiformes | | Petromyzontidae | *Ichthyomyzon fossor* | | 5to25 |
| Cephalaspidomorphi | Petromyzontiformes | | Petromyzontidae | *Ichthyomyzon unicuspis* | | >25 |
| Cephalaspidomorphi | Petromyzontiformes | | Petromyzontidae | *Lethenteron appendix* | | >25 |
| Cephalaspidomorphi | Petromyzontiformes | | Petromyzontidae | ***Petromyzon marinus*** | | >25 |
| Reptilia | Squametia | | Colubridae | Elaphe vulpina | | <5 |
| Reptilia | Squametia | | Colubridae | Lampropeltis triangulum | | <5 |
| Reptilia | Squametia | | Colubridae | Nerodia sipedon | | 5to25 |
| Reptilia | Squametia | | Colubridae | Regina grahamii | | <5 |
| Reptilia | Squametia | | Colubridae | Regina septemvittata | | <5 |
| Reptilia | Squametia | | Colubridae | Storeria dekayi | | 5to25 |
| Reptilia | Squametia | | Colubridae | Thamnophis butleri | | <5 |
| Reptilia | Squametia | | Colubridae | Thamnophis proximus proximus | | 5to25 |
| Reptilia | Squametia | | Colubridae | Thamnophis radix | | <5 |
| Reptilia | Squametia | | Colubridae | Thamnophis sauritus | | 5to25 |
| Reptilia | Squametia | | Colubridae | Thamnophis sirtalis | | 5to25 |
| Reptilia | Squametia | | Viperidae | Sistrurus catenatus | | 5to25 |
| Reptilia | Testudines | | Chelydridae | Chelydra serpentina | | 5to25 |
| Reptilia | Testudines | | Emydidae | Chrysemys picta | | 5to25 |
| Reptilia | Testudines | | Emydidae | Clemmys guttata | | 5to25 |
| Reptilia | Testudines | | Emydidae | Emydoidea blandingii | | 5to25 |
| Reptilia | Testudines | | Emydidae | Glyptemys insculpta | | <5 |
| Reptilia | Testudines | | Emydidae | Glyptemys muhlenbergii | | <5 |
| Reptilia | Testudines | | Emydidae | Graptemys geographica | | <5 |
| Reptilia | Testudines | | Emydidae | Trachemys scripta | | 5to25 |
| Reptilia | Testudines | | Kinosternidae | Sternotherus odoratus | | <5 |
| Reptilia | Testudines | | Trionychidae | Apalone spinifera | | 5to25 |
| **Phylum *Cnidaria*** | | | | | | |
| Hydrozoa | Anthoathecatae | | Hydridae | Hydra americana | | lacking |
| Hydrozoa | Anthoathecatae | | Oceanidae | **Cordylophora caspia ** | | lacking |
| Hydrozoa | Limnimedusae | | Craspedacusta | **Craspedacusta sowerbyi** | | 5to25 |
| **Phylum *Kamptozoa*** | | | | | | |
| Entoprocta | Solitaria | | Barentsiidae | Urnatella gracilis  (in BOLD as Barentsia gracilis) | | <5 |
| **Phylum *Mollusca*** | | | | | | |
| Bivalvia | Veneroida | | Corbiculidae | **Corbicula fluminea** | | 5to25 |
| Gastropoda | Architaenioglossa | | Viviparidae | Campeloma decisum | | zero |
| Gastropoda | Architaenioglossa | | Viviparidae | Campeloma rufum | | lacking |
| Gastropoda | Architaenioglossa | | Viviparidae | **Cipangopaludina chinensis** | | <5 |
| Gastropoda | Architaenioglossa | | Viviparidae | **Cipangopaludina japonica** | | <5 |
| Gastropoda | Architaenioglossa | | Viviparidae | **Viviparus georgianus** | | <5 |
| Gastropoda | Basommatophora | | Ancylidae | Ferrissia parallela | | lacking |
| Gastropoda | Basommatophora | | Ancylidae | Ferrissia parallelus | | lacking |
| Gastropoda | Basommatophora | | Ancylidae | Ferrissia rivularis | | <5 |
| Gastropoda | Basommatophora | | Ancylidae | Laevapex fuscus | | <5 |
| Gastropoda | Basommatophora | | Lymnaeidae | Acella haldemani | | lacking |
| Gastropoda | Basommatophora | | Lymnaeidae | Bulimnaea megasoma | | lacking |
| Gastropoda | Basommatophora | | Lymnaeidae | Fossaria dalli | | lacking |
| Gastropoda | Basommatophora | | Lymnaeidae | Fossaria decampi | | lacking |
| Gastropoda | Basommatophora | | Lymnaeidae | Fossaria humilis | | lacking |
| Gastropoda | Basommatophora | | Lymnaeidae | Fossaria modicella | | 5to25 |
| Gastropoda | Basommatophora | | Lymnaeidae | Fossaria obrussa | | lacking |
| Gastropoda | Basommatophora | | Lymnaeidae | Fossaria parva | | 5to25 |
| Gastropoda | Basommatophora | | Lymnaeidae | Lymnaea auricularia | | lacking |
| Gastropoda | Basommatophora | | Lymnaeidae | Lymnaea stagnalis | | >25 |
| Gastropoda | Basommatophora | | Lymnaeidae | Pseudosuccinea columella | | 5to25 |
| Gastropoda | Basommatophora | | Lymnaeidae | **Radix auricularia** | | 5to25 |
| Gastropoda | Basommatophora | | Lymnaeidae | Stagnicola caperata | | lacking |
| Gastropoda | Basommatophora | | Lymnaeidae | Stagnicola catascopium | | lacking |
| Gastropoda | Basommatophora | | Lymnaeidae | Stagnicola elodes | | >25 |
| Gastropoda | Basommatophora | | Lymnaeidae | Stagnicola emerginata | | lacking |
| Gastropoda | Basommatophora | | Lymnaeidae | Stagnicola exilis | | lacking |
| Gastropoda | Basommatophora | | Lymnaeidae | Stagnicola reflexa | | lacking |
| Gastropoda | Basommatophora | | Physidae | Physa heterostropha | | 5to25 |
| Gastropoda | Basommatophora | | Physidae | Physella gyrina | | 5to25 |
| Gastropoda | Basommatophora | | Physidae | Physella integra | | lacking |
| Gastropoda | Basommatophora | | Physidae | Physella vinosa | | lacking |
| Gastropoda | Basommatophora | | Planorbidae | Gyraulus altissimus | | lacking |
| Gastropoda | Basommatophora | | Planorbidae | Gyraulus circumstriatus | | 5to25 |
| Gastropoda | Basommatophora | | Planorbidae | Gyraulus deflectus | | lacking |
| Gastropoda | Basommatophora | | Planorbidae | Gyraulus parvus | | <5 |
| Gastropoda | Basommatophora | | Planorbidae | Helisoma anceps | | <5 |
| Gastropoda | Basommatophora | | Planorbidae | Planorbella companulatum | | lacking |
| Gastropoda | Basommatophora | | Planorbidae | Planorbella corpulenta | | lacking |
| Gastropoda | Basommatophora | | Planorbidae | Planorbella pilbryi | | lacking |
| Gastropoda | Basommatophora | | Planorbidae | Planorbella trivolvis | | <5 |
| Gastropoda | Basommatophora | | Planorbidae | Planorbula armigera | | <5 |
| Gastropoda | Basommatophora | | Planorbidae | Promenetus exacuous | | 5to25 |
| Gastropoda | Heterostropha | | Valvatidae | Valvata bicarinata | | lacking |
| Gastropoda | Heterostropha | | Valvatidae | Valvata lewisi | | lacking |
| Gastropoda | Heterostropha | | Valvatidae | Valvata perdepressa | | lacking |
| Gastropoda | Heterostropha | | Valvatidae | **Valvata piscinalis** | | 5to25 |
| Gastropoda | Heterostropha | | Valvatidae | Valvata sincera (in BOLD as Valvata sincera helicoidea) | | 5to25 |
| Gastropoda | Heterostropha | | Valvatidae | Valvata tricarinata | | lacking |
| Gastropoda | Neotaenioglossa | | Bithyniidae | Amnicola walkeri | | lacking |
| Gastropoda | Neotaenioglossa | | Bithyniidae | **Bithynia tentaculata** | | 5to25 |
| Gastropoda | Neotaenioglossa | | Hydrobiidae | Amnicola limosus | | 5to25 |
| Gastropoda | Neotaenioglossa | | Hydrobiidae | Cincinnatia cincinnatiensis | | lacking |
| Gastropoda | Neotaenioglossa | | Hydrobiidae | Cincinnatia integra | | <5 |
| Gastropoda | Neotaenioglossa | | Hydrobiidae | **Gillia altilis** | | lacking |
| Gastropoda | Neotaenioglossa | | Hydrobiidae | Hoyia sheldoni | | lacking |
| Gastropoda | Neotaenioglossa | | Hydrobiidae | Marstonia decepta | | lacking |
| Gastropoda | Neotaenioglossa | | Hydrobiidae | Marstonia lustrica | | <5 |
| Gastropoda | Neotaenioglossa | | Hydrobiidae | **Potamopyrgus antipodarum** | | >25 |
| Gastropoda | Neotaenioglossa | | Hydrobiidae | Probythinella emarginata | | <5 |
| Gastropoda | Neotaenioglossa | | Hydrobiidae | Probythinella lacustris | | lacking |
| Gastropoda | Neotaenioglossa | | Hydrobiidae | Pyrgulopsis lustrica | | lacking |
| Gastropoda | Neotaenioglossa | | Hydrobiidae | Somatogyrus subglobosus | | lacking |
| Gastropoda | Neotaenioglossa | | Pleuroceridae | Elimia livescens | | <5 |
| Gastropoda | Neotaenioglossa | | Pleuroceridae | **Elimia virginica** | | lacking |
| Gastropoda | Neotaenioglossa | | Pleuroceridae | Goniobasis livescens | | lacking |
| Gastropoda | Neotaenioglossa | | Pleuroceridae | Pleurocera acuta | | lacking |
| Gastropoda | Stylommatophora | | Succineidae | Oxyloma retusum  (in BOLD as O. retusa) | | lacking |
| Pelecypoda | Unionoida | | Unionidae | Actinonaias ligamentina | | 5to25 |
| Pelecypoda | Unionoida | | Unionidae | Alasmidonta marginata | | <5 |
| Pelecypoda | Unionoida | | Unionidae | Alasmidonta undulata | | lacking |
| Pelecypoda | Unionoida | | Unionidae | Alasmidonta viridis | | lacking |
| Pelecypoda | Unionoida | | Unionidae | Amblema plicata | | 5to25 |
| Pelecypoda | Unionoida | | Unionidae | Anodontoides ferussacianus | | zero |
| Pelecypoda | Unionoida | | Unionidae | Cyclonaias tuberculata | | 5to25 |
| Pelecypoda | Unionoida | | Unionidae | Elliptio complanata | | >25 |
| Pelecypoda | Unionoida | | Unionidae | Elliptio dilatata | | 5to25 |
| Pelecypoda | Unionoida | | Unionidae | Epioblasma torulosa | | 5to25 |
| Pelecypoda | Unionoida | | Unionidae | Epioblasma triquetra | | 5to25 |
| Pelecypoda | Unionoida | | Unionidae | Fusconaia flava | | 5to25 |
| Pelecypoda | Unionoida | | Unionidae | Lampsilis cardium | | <5 |
| Pelecypoda | Unionoida | | Unionidae | Lampsilis fasciola | | <5 |
| Pelecypoda | Unionoida | | Unionidae | Lampsilis ovata | | <5 |
| Pelecypoda | Unionoida | | Unionidae | Lampsilis radiata | | 5to25 |
| Pelecypoda | Unionoida | | Unionidae | Lampsilis siliquoidea | | >25 |
| Pelecypoda | Unionoida | | Unionidae | Lasmigona complanata | | lacking |
| Pelecypoda | Unionoida | | Unionidae | Lasmigona compressa | | 5to25 |
| Pelecypoda | Unionoida | | Unionidae | Lasmigona costata | | 5to25 |
| Pelecypoda | Unionoida | | Unionidae | **Lasmigona subviridis** | | lacking |
| Pelecypoda | Unionoida | | Unionidae | Leptodea fragilis | | 5to25 |
| Pelecypoda | Unionoida | | Unionidae | Ligumia nasuta | | <5 |
| Pelecypoda | Unionoida | | Unionidae | Ligumia recta | | 5to25 |
| Pelecypoda | Unionoida | | Unionidae | Obliquaria reflexa | | 5to25 |
| Pelecypoda | Unionoida | | Unionidae | Obovaria olivaria | | >25 |
| Pelecypoda | Unionoida | | Unionidae | Obovaria subrotunda | | 5to25 |
| Pelecypoda | Unionoida | | Unionidae | Pleurobema coccineum | | lacking |
| Pelecypoda | Unionoida | | Unionidae | Pleurobema cordatum | | <5 |
| Pelecypoda | Unionoida | | Unionidae | Pleurobema sintoxia | | 5to25 |
| Pelecypoda | Unionoida | | Unionidae | Potamilus alatus | | 5to25 |
| Pelecypoda | Unionoida | | Unionidae | Potamilus ohiensis | | <5 |
| Pelecypoda | Unionoida | | Unionidae | Ptychobranchus fasciolaris | | <5 |
| Pelecypoda | Unionoida | | Unionidae | Pyganodon cataracta | | 5to25 |
| Pelecypoda | Unionoida | | Unionidae | Pyganodon grandis | | 5to25 |
| Pelecypoda | Unionoida | | Unionidae | Quadrula nodulata | | <5 |
| Pelecypoda | Unionoida | | Unionidae | Quadrula pustulosa | | <5 |
| Pelecypoda | Unionoida | | Unionidae | Quadrula quadrula | | 5to25 |
| Pelecypoda | Unionoida | | Unionidae | Simpsonaias ambigua | | lacking |
| Pelecypoda | Unionoida | | Unionidae | Strophitus undulatus | | 5to25 |
| Pelecypoda | Unionoida | | Unionidae | Toxolasma parvum (in BOLD as T. parvus) | | >25 |
| Pelecypoda | Unionoida | | Unionidae | Truncilla donaciformis | | <5 |
| Pelecypoda | Unionoida | | Unionidae | Truncilla truncata | | <5 |
| Pelecypoda | Unionoida | | Unionidae | Utterbackia imbecillis | | 5to25 |
| Pelecypoda | Unionoida | | Unionidae | Villosa fabalis | | <5 |
| Pelecypoda | Unionoida | | Unionidae | Villosa iris | | 5to25 |
| Pelecypoda | Veneroida | | Dreisseniidae | **Dreissena bugensis** | | 5to25 |
| Pelecypoda | Veneroida | | Dreisseniidae | **Dreissena polymorpha** | | >25 |
| Pelecypoda | Veneroida | | Pisidiidae | Musculium lacustre | | lacking |
| Pelecypoda | Veneroida | | Pisidiidae | Musculium partumeium | | lacking |
| Pelecypoda | Veneroida | | Pisidiidae | Musculium securis | | lacking |
| Pelecypoda | Veneroida | | Pisidiidae | Musculium transversum | | lacking |
| Pelecypoda | Veneroida | | Pisidiidae | Pisidium adamsi | | lacking |
| Pelecypoda | Veneroida | | Pisidiidae | **Pisidium amnicum ** | | lacking |
| Pelecypoda | Veneroida | | Pisidiidae | Pisidium casertanum | | zero |
| Pelecypoda | Veneroida | | Pisidiidae | Pisidium compressum | | lacking |
| Pelecypoda | Veneroida | | Pisidiidae | Pisidium conventus | | lacking |
| Pelecypoda | Veneroida | | Pisidiidae | Pisidium dubium | | lacking |
| Pelecypoda | Veneroida | | Pisidiidae | Pisidium equilaterale | | lacking |
| Pelecypoda | Veneroida | | Pisidiidae | Pisidium fallax | | lacking |
| Pelecypoda | Veneroida | | Pisidiidae | Pisidium ferrugineum | | lacking |
| Pelecypoda | Veneroida | | Pisidiidae | **Pisidium henslowanum ** | | zero |
| Pelecypoda | Veneroida | | Pisidiidae | Pisidium idahoense | | lacking |
| Pelecypoda | Veneroida | | Pisidiidae | Pisidium insigne | | lacking |
| Pelecypoda | Veneroida | | Pisidiidae | Pisidium lilljeborgi | | lacking |
| Pelecypoda | Veneroida | | Pisidiidae | Pisidium milium | | zero |
| Pelecypoda | Veneroida | | Pisidiidae | **Pisidium moitessierianum ** | | lacking |
| Pelecypoda | Veneroida | | Pisidiidae | Pisidium nitidum | | <5 |
| Pelecypoda | Veneroida | | Pisidiidae | Pisidium punctatum | | lacking |
| Pelecypoda | Veneroida | | Pisidiidae | Pisidium subtruncatum | | zero |
| Pelecypoda | Veneroida | | Pisidiidae | **Pisidium supinum ** | | zero |
| Pelecypoda | Veneroida | | Pisidiidae | Pisidium variabile | | lacking |
| Pelecypoda | Veneroida | | Pisidiidae | Pisidium ventricosum | | <5 |
| Pelecypoda | Veneroida | | Pisidiidae | Pisidium walkeri | | lacking |
| Pelecypoda | Veneroida | | Pisidiidae | **Sphaerium corneum ** | | zero |
| Pelecypoda | Veneroida | | Pisidiidae | Sphaerium nitidum | | lacking |
| Pelecypoda | Veneroida | | Pisidiidae | Sphaerium occidentale | | lacking |
| Pelecypoda | Veneroida | | Pisidiidae | Sphaerium rhomboideum | | lacking |
| Pelecypoda | Veneroida | | Pisidiidae | Sphaerium simile | | lacking |
| Pelecypoda | Veneroida | | Pisidiidae | Sphaerium striatinum | | <5 |
| **Phylum *Nematomorpha*** | | | | | | |
| Gordioida | Gordea | Gordiidae | | Gordius robustus | | lacking |
| **Phylum *Nemertea*** | | | | | | |
| Enopla | Hoplonemertea | Tetrastemmatidae | | Prostoma graecense | | <5 |
| **Phylum *Platyhelminthes*** | | | | | | |
| Trematoda | Monopisthocotylea | | Dactylogyridae | Dactylogyrus banghami | | lacking |
| Trematoda | Monopisthocotylea | | Dactylogyridae | Dactylogyrus vastator | | lacking |
| Trematoda | Strigeidida | | Diplostomatidae | **Neascus brevicaudatus** | | lacking |
| Trepaxonemata | Neoophora | | Dendrocoelidae | Procotyla fluviatilis | | lacking |
| Trepaxonemata | Neoophora | | Dugesiidae | Cura foremanii | | lacking |
| Trepaxonemata | Neoophora | | Plagiostomidae | Hydrolimax grisea | | lacking |
| Trepaxonemata | Neoophora | | Planariidae | **Dugesia polychroa** | | lacking |
| Trepaxonemata | Neoophora | | Planariidae | Dugesia tigrina | | lacking |
| Trepaxonemata | Neoophora | | Planariidae | Hymanella retenuova | | lacking |
| **Phylum *Porifera*** | | | | | | |
| Demospongiae | Haplosclerida | | Spongillidae | Ephydatia mulleri | | 5to25 |
| Demospongiae | Haplosclerida | | Spongillidae | Eunapius fragilis | | <5 |
| Demospongiae | Haplosclerida | | Spongillidae | Spongilla lacustris | | <5 |
| **Phylum *Rotifera*** | | | | | | |
| Bdelloidea | Philodinidae | | Rotaria | *Rotaria neptunia* | <5 | |
| Monogonta | Collothecaceae | | Collothecidae | *Collotheca mutabilis* | genus | |
| Monogonta | Collothecaceae | | Collothecidae | *Collotheca pelagica* | genus | |
| Monogonta | Flosculariaceae | | Conochilidae | *Conochiloides dossuarius* | lacking | |
| Monogonta | Flosculariaceae | | Conochilidae | *Conochilus hippocrepis* | lacking | |
| Monogonta | Flosculariaceae | | Conochilidae | *Conochilus unicornis* | <5 | |
| Monogonta | Flosculariaceae | | Filiniidae | *Filinia longiseta* | <5 | |
| Monogonta | Flosculariaceae | | Filiniidae | *Filinia terminalis* (in BOLD as Filinia cf. terminalis) | <5 | |
| Monogonta | Flosculariaceae | | Hexarthridae | *Hexarthra mira* | zero | |
| Monogonta | Flosculariaceae | | Testudinellidae | *Pompholyx sulcata* | lacking | |
| Monogonta | Flosculariaceae | | Testudinellidae | *Testudinella patina* | 5to25 | |
| Monogonta | Ploima | | Asplanchnidae | *Asplanchna brightwelli* | <5 | |
| Monogonta | Ploima | | Asplanchnidae | *Asplanchna giroidi* | lacking | |
| Monogonta | Ploima | | Asplanchnidae | *Asplanchna herricki* | lacking | |
| Monogonta | Ploima | | Asplanchnidae | *Asplanchna priodonta* | 5to25 | |
| Monogonta | Ploima | | Asplanchnidae | *Asplanchna sieboldi* | 5to25 | |
| Monogonta | Ploima | | Brachionidae | *Anuraeopsis fissa* | <5 | |
| Monogonta | Ploima | | Brachionidae | *Anuraeopsis navicula* | lacking | |
| Monogonta | Ploima | | Brachionidae | *Brachionus angularis* | 5to25 | |
| Monogonta | Ploima | | Brachionidae | *Brachionus bidentata*  (in BOLD as B. bidentatus) | 5to25 | |
| Monogonta | Ploima | | Brachionidae | *Brachionus budapestinensis* | <5 | |
| Monogonta | Ploima | | Brachionidae | *Brachionus calyciflorus* | >25 | |
| Monogonta | Ploima | | Brachionidae | *Brachionus caudatus* | <5 | |
| Monogonta | Ploima | | Brachionidae | *Brachionus diversicornis* | lacking | |
| Monogonta | Ploima | | Brachionidae | *Brachionus havanaensis* | 5to25 | |
| Monogonta | Ploima | | Brachionidae | *Brachionus quadridentatus* | 5to25 | |
| Monogonta | Ploima | | Brachionidae | *Brachionus rubens* | <5 | |
| Monogonta | Ploima | | Brachionidae | *Brachionus urceolaris* | 5to25 | |
| Monogonta | Ploima | | Brachionidae | *Brachionus variabilis* | 5to25 | |
| Monogonta | Ploima | | Brachionidae | *Colurella obtusa* | zero | |
| Monogonta | Ploima | | Brachionidae | *Colurella uncinata* | zero | |
| Monogonta | Ploima | | Brachionidae | *Epiphanes clavulata* | zero | |
| Monogonta | Ploima | | Brachionidae | *Epiphanes pelagica* | lacking | |
| Monogonta | Ploima | | Brachionidae | *Kellicottia bostoniensis* | 5to25 | |
| Monogonta | Ploima | | Brachionidae | *Kellicottia longispina* | lacking | |
| Monogonta | Ploima | | Brachionidae | *Keratella cochlearis* | >25 | |
| Monogonta | Ploima | | Brachionidae | *Keratella crassa* | lacking | |
| Monogonta | Ploima | | Brachionidae | *Keratella earlinae* | lacking | |
| Monogonta | Ploima | | Brachionidae | *Keratella hiemalis* | <5 | |
| Monogonta | Ploima | | Brachionidae | *Keratella irregularis* | lacking | |
| Monogonta | Ploima | | Brachionidae | *Keratella quadrata* | 5to25 | |
| Monogonta | Ploima | | Brachionidae | *Keratella serrulata* | lacking | |
| Monogonta | Ploima | | Brachionidae | *Keratella taurocephala* | lacking | |
| Monogonta | Ploima | | Brachionidae | *Keratella valga* | <5 | |
| Monogonta | Ploima | | Brachionidae | *Lepadella acuminata* | zero | |
| Monogonta | Ploima | | Brachionidae | *Lepadella aspida* | lacking | |
| Monogonta | Ploima | | Brachionidae | *Lepadella ehrenbergi* | lacking | |
| Monogonta | Ploima | | Brachionidae | *Lepadella ovalis* | zero | |
| Monogonta | Ploima | | Brachionidae | *Lepadella patella* | <5 | |
| Monogonta | Ploima | | Brachionidae | *Lepadella rhomboides* | zero | |
| Monogonta | Ploima | | Brachionidae | *Lepadella triptera* | zero | |
| Monogonta | Ploima | | Brachionidae | *Lophocharis oxysternon* | lacking | |
| Monogonta | Ploima | | Brachionidae | *Lophocharis salpina* | <5 | |
| Monogonta | Ploima | | Brachionidae | *Macrochaetus collinsi* | 5to25 | |
| Monogonta | Ploima | | Brachionidae | *Macrochaetus serica* | lacking | |
| Monogonta | Ploima | | Brachionidae | *Macrochaetus subquadratus* | lacking | |
| Monogonta | Ploima | | Brachionidae | *Mikrocodides chlaena* | lacking | |
| Monogonta | Ploima | | Brachionidae | *Mytilina trigona* | lacking | |
| Monogonta | Ploima | | Brachionidae | *Mytilina ventralis* | 5to25 | |
| Monogonta | Ploima | | Brachionidae | *Notholca acuminata* | <5 | |
| Monogonta | Ploima | | Brachionidae | *Notholca caudata* | lacking | |
| Monogonta | Ploima | | Brachionidae | *Notholca foliacea* | lacking | |
| Monogonta | Ploima | | Brachionidae | *Notholca labis* | lacking | |
| Monogonta | Ploima | | Brachionidae | *Notholca laurentiae* | lacking | |
| Monogonta | Ploima | | Brachionidae | *Notholca michiganensis* | lacking | |
| Monogonta | Ploima | | Brachionidae | *Notholca squamula* | lacking | |
| Monogonta | Ploima | | Brachionidae | *Notholca striata* | lacking | |
| Monogonta | Ploima | | Brachionidae | *Platyias patulus* | lacking | |
| Monogonta | Ploima | | Brachionidae | *Platyias quadricornis* | 5to25 | |
| Monogonta | Ploima | | Brachionidae | *Squatinella mutica* | <5 | |
| Monogonta | Ploima | | Brachionidae | *Squatinella rostrum* | zero | |
| Monogonta | Ploima | | Brachionidae | *Trichotria pocillum* | lacking | |
| Monogonta | Ploima | | Brachionidae | *Trichotria tetractis* | 5to25 | |
| Monogonta | Ploima | | Brachionidae | *Wolga spinifera* | lacking | |
| Monogonta | Ploima | | Dicranophoridae | *Aspelta aper* | lacking | |
| Monogonta | Ploima | | Dicranophoridae | *Dicranophorus caudatus* | lacking | |
| Monogonta | Ploima | | Dicranophoridae | *Dicranophorus forcipatus* | lacking | |
| Monogonta | Ploima | | Dicranophoridae | *Dicranophorus grandis* | lacking | |
| Monogonta | Ploima | | Dicranophoridae | *Dicranophorus mesotis* | lacking | |
| Monogonta | Ploima | | Dicranophoridae | *Dicranophorus tegillus* | lacking | |
| Monogonta | Ploima | | Dicranophoridae | *Encentrum felis* | lacking | |
| Monogonta | Ploima | | Dicranophoridae | *Encentrum saundersiae* | lacking | |
| Monogonta | Ploima | | Dicranophoridae | *Wierzejkiella velox*  (not found in ITIS) | lacking | |
| Monogonta | Ploima | | Euchlanidae | *Euchlanis alata* | <5 | |
| Monogonta | Ploima | | Euchlanidae | *Euchlanis calpidia* | lacking | |
| Monogonta | Ploima | | Euchlanidae | *Euchlanis deflexa* | <5 | |
| Monogonta | Ploima | | Euchlanidae | *Euchlanis dilatata* | 5to25 | |
| Monogonta | Ploima | | Euchlanidae | *Euchlanis meneta* | <5 | |
| Monogonta | Ploima | | Euchlanidae | *Euchlanis oropha* | lacking | |
| Monogonta | Ploima | | Euchlanidae | *Euchlanis parva* | lacking | |
| Monogonta | Ploima | | Euchlanidae | *Euchlanis pellucida* | lacking | |
| Monogonta | Ploima | | Euchlanidae | *Euchlanis proxima* | lacking | |
| Monogonta | Ploima | | Euchlanidae | *Euchlanis triquetra* | lacking | |
| Monogonta | Ploima | | Gastropodidae | *Ascomorpha ecaudis* | lacking | |
| Monogonta | Ploima | | Gastropodidae | *Ascomorpha ovalis* | 5to25 | |
| Monogonta | Ploima | | Gastropodidae | *Ascomorpha saltans* | lacking | |
| Monogonta | Ploima | | Gastropodidae | *Gastropus hyptopus* | lacking | |
| Monogonta | Ploima | | Gastropodidae | *Gastropus minor* | lacking | |
| Monogonta | Ploima | | Gastropodidae | *Gastropus stylifer* | lacking | |
| Monogonta | Ploima | | Lecanidae | *Lecane arcula* | <5 | |
| Monogonta | Ploima | | Lecanidae | *Lecane crepida* | 5to25 | |
| Monogonta | Ploima | | Lecanidae | *Lecane curvicornis* | <5 | |
| Monogonta | Ploima | | Lecanidae | *Lecane depressa* | lacking | |
| Monogonta | Ploima | | Lecanidae | *Lecane flexilis* | zero | |
| Monogonta | Ploima | | Lecanidae | *Lecane hastata* | <5 | |
| Monogonta | Ploima | | Lecanidae | *Lecane inermis* | lacking | |
| Monogonta | Ploima | | Lecanidae | *Lecane inopinata* | lacking | |
| Monogonta | Ploima | | Lecanidae | *Lecane leontina* | 5to25 | |
| Monogonta | Ploima | | Lecanidae | *Lecane ludwigii* | 5to25 | |
| Monogonta | Ploima | | Lecanidae | *Lecane luna* | 5to25 | |
| Monogonta | Ploima | | Lecanidae | *Lecane mira* | lacking | |
| Monogonta | Ploima | | Lecanidae | *Lecane mucronata* | lacking | |
| Monogonta | Ploima | | Lecanidae | *Lecane ohioensis* | lacking | |
| Monogonta | Ploima | | Lecanidae | *Lecane stokesi* | lacking | |
| Monogonta | Ploima | | Lecanidae | *Lecane tenuiseta* | lacking | |
| Monogonta | Ploima | | Lecanidae | *Lecane tudicola* | lacking | |
| Monogonta | Ploima | | Lecanidae | *Lecane ungulata* | zero | |
| Monogonta | Ploima | | Lecanidae | *Monostyla bulla* | lacking | |
| Monogonta | Ploima | | Lecanidae | *Monostyla closterocerca* | lacking | |
| Monogonta | Ploima | | Lecanidae | *Monostyla copeis* | lacking | |
| Monogonta | Ploima | | Lecanidae | *Monostyla cornuta* | lacking | |
| Monogonta | Ploima | | Lecanidae | *Monostyla crenata* | lacking | |
| Monogonta | Ploima | | Lecanidae | *Monostyla hamata* | lacking | |
| Monogonta | Ploima | | Lecanidae | *Monostyla lunaris* | lacking | |
| Monogonta | Ploima | | Lecanidae | *Monostyla obtusa* | lacking | |
| Monogonta | Ploima | | Lecanidae | *Monostyla pyriformis* | lacking | |
| Monogonta | Ploima | | Lecanidae | *Monostyla quadridentata* | lacking | |
| Monogonta | Ploima | | Lecanidae | *Monostyla rhopalura*  (not found in ITIS) | lacking | |
| Monogonta | Ploima | | Lecanidae | *Monostyla rugosa* | lacking | |
| Monogonta | Ploima | | Lecanidae | *Monostyla stenroosi* | lacking | |
| Monogonta | Ploima | | Lindidae | *Lindia torulosa*  (not found in ITIS) | lacking | |
| Monogonta | Ploima | | Notommatidae | *Cephalodella auriculata* | lacking | |
| Monogonta | Ploima | | Notommatidae | *Cephalodella crassipes* | lacking | |
| Monogonta | Ploima | | Notommatidae | *Cephalodella forficula* | zero | |
| Monogonta | Ploima | | Notommatidae | *Cephalodella gibba* | <5 | |
| Monogonta | Ploima | | Notommatidae | *Cephalodella gracilis* | lacking | |
| Monogonta | Ploima | | Notommatidae | *Cephalodella intuta* | lacking | |
| Monogonta | Ploima | | Notommatidae | *Cephalodella megalocephala* | lacking | |
| Monogonta | Ploima | | Notommatidae | *Cephalodella rotunda*  (not found in ITIS) | lacking | |
| Monogonta | Ploima | | Notommatidae | *Itura aurita* | lacking | |
| Monogonta | Ploima | | Notommatidae | *Monommata grandis* | lacking | |
| Monogonta | Ploima | | Notommatidae | *Monommata longiseta* | lacking | |
| Monogonta | Ploima | | Notommatidae | *Notommata aurita* | lacking | |
| Monogonta | Ploima | | Notommatidae | *Notommata brachyota*  (not found in ITIS) | lacking | |
| Monogonta | Ploima | | Notommatidae | *Notommata cerberus* | lacking | |
| Monogonta | Ploima | | Notommatidae | *Notommata collaris* | lacking | |
| Monogonta | Ploima | | Notommatidae | *Notommata copeus* | lacking | |
| Monogonta | Ploima | | Notommatidae | *Notommata cyrtopus* | lacking | |
| Monogonta | Ploima | | Notommatidae | *Notommata lenis* | lacking | |
| Monogonta | Ploima | | Notommatidae | *Notommata pachyura* | lacking | |
| Monogonta | Ploima | | Notommatidae | *Notommata tripus* | lacking | |
| Monogonta | Ploima | | Notommatidae | *Scaridium longicaudum* | lacking | |
| Monogonta | Ploima | | Notommatidae | *Taphrocampa annulosa* | lacking | |
| Monogonta | Ploima | | Notommatidae | *Taphrocampa selenura* | lacking | |
| Monogonta | Ploima | | Proalidae | *Proales decipiens* | lacking | |
| Monogonta | Ploima | | Proalidae | *Proales parasita* | lacking | |
| Monogonta | Ploima | | Proalidae | *Proales sordida* | lacking | |
| Monogonta | Ploima | | Proalidae | *Proales werneckii* | lacking | |
| Monogonta | Ploima | | Synchaetidae | *Ploesoma hudsoni* | lacking | |
| Monogonta | Ploima | | Synchaetidae | *Ploesoma lenticulare* | lacking | |
| Monogonta | Ploima | | Synchaetidae | *Ploesoma truncatum* | lacking | |
| Monogonta | Ploima | | Synchaetidae | *Polyarthra dissimulans* | lacking | |
| Monogonta | Ploima | | Synchaetidae | *Polyarthra dolichoptera* | 5to25 | |
| Monogonta | Ploima | | Synchaetidae | *Polyarthra euryptera* | lacking | |
| Monogonta | Ploima | | Synchaetidae | *Polyarthra longiremis*  (not found in ITIS) | lacking | |
| Monogonta | Ploima | | Synchaetidae | *Polyarthra major* | lacking | |
| Monogonta | Ploima | | Synchaetidae | *Polyarthra remata* | 5to25 | |
| Monogonta | Ploima | | Synchaetidae | *Polyarthra vulgaris* | lacking | |
| Monogonta | Ploima | | Synchaetidae | *Synchaeta asymmetrica* | lacking | |
| Monogonta | Ploima | | Synchaetidae | *Synchaeta grandis* | 5to25 | |
| Monogonta | Ploima | | Synchaetidae | *Synchaeta kitina* | >25 | |
| Monogonta | Ploima | | Synchaetidae | *Synchaeta lakowitziana* | 5to25 | |
| Monogonta | Ploima | | Synchaetidae | *Synchaeta oblonga* | 5to25 | |
| Monogonta | Ploima | | Synchaetidae | *Synchaeta pectinata* | >25 | |
| Monogonta | Ploima | | Synchaetidae | *Synchaeta stylata* | zero | |
| Monogonta | Ploima | | Trichocercidae | *Ascomorphella volvocicola* | lacking | |
| Monogonta | Ploima | | Trichocercidae | *Trichocerca bicristata* | zero | |
| Monogonta | Ploima | | Trichocercidae | *Trichocerca brachyura* | lacking | |
| Monogonta | Ploima | | Trichocercidae | *Trichocerca capucina* (in BOLD as Trichocerca cf. capucina) | <5 | |
| Monogonta | Ploima | | Trichocercidae | *Trichocerca cylindrica* | lacking | |
| Monogonta | Ploima | | Trichocercidae | *Trichocerca elongata* | lacking | |
| Monogonta | Ploima | | Trichocercidae | *Trichocerca iernis* | lacking | |
| Monogonta | Ploima | | Trichocercidae | *Trichocerca insignis* | lacking | |
| Monogonta | Ploima | | Trichocercidae | *Trichocerca insolens* | lacking | |
| Monogonta | Ploima | | Trichocercidae | *Trichocerca lata* | lacking | |
| Monogonta | Ploima | | Trichocercidae | *Trichocerca longiseta* | <5 | |
| Monogonta | Ploima | | Trichocercidae | *Trichocerca lophoessa* | lacking | |
| Monogonta | Ploima | | Trichocercidae | *Trichocerca mucosa* | lacking | |
| Monogonta | Ploima | | Trichocercidae | *Trichocerca multicrinis* | lacking | |
| Monogonta | Ploima | | Trichocercidae | *Trichocerca porcellus* | zero | |
| Monogonta | Ploima | | Trichocercidae | *Trichocerca pusilla* | <5 | |
| Monogonta | Ploima | | Trichocercidae | *Trichocerca rattus* | lacking | |
| Monogonta | Ploima | | Trichocercidae | *Trichocerca rousseleti* | lacking | |
| Monogonta | Ploima | | Trichocercidae | *Trichocerca similis* | 5to25 | |
| Monogonta | Ploima | | Trichocercidae | *Trichocerca stylata* | 5to25 | |
| Monogonta | Ploima | | Trichocercidae | *Trichocerca sulcata* | lacking | |
| Monogonta | Ploima | | Trichocercidae | *Trichocerca tenuior* | <5 | |
| Monogonta | Ploima | | Trichocercidae | *Trichocerca tigris* | lacking | |
| Monogonta | Ploima | | Trichocercidae | *Trichocerca weberi* | lacking | |
| Monogonta | Ploima | | Trichotriidae | *Trichotria tetractis* | 5to25 | |
| Monogonta | Ploima | | Tylotrochidae | *Tylotrocha monopus* | lacking | |
